# The Punctum Fixum-Punctum Mobile Model: A Neuromuscular Principle for Efficient Movement Generation?

**DOI:** 10.1371/journal.pone.0120193

**Published:** 2015-03-30

**Authors:** Christoph von Laßberg, Walter Rapp

**Affiliations:** 1 Institute of General Kinesiology and Athletics Training, University of Leipzig, Leipzig, Germany; 2 Medical Clinic, Department of Sports Medicine, University of Tübingen, Tübingen, Germany; The University of Queensland, AUSTRALIA

## Abstract

According to the “punctum fixum–punctum mobile model” that was introduced in prior studies, for generation of the most effective intentional acceleration of a body part the intersegmental neuromuscular onset succession has to spread successively from the rotation axis (punctum fixum) toward the body part that shall be accelerated (punctum mobile). The aim of the present study was to investigate whether this principle is, indeed, fundamental for *any* kind of efficient rotational accelerations in general, independent of the kind of movements, type of rotational axis, the current body position, or movement direction. Neuromuscular onset succession was captured by surface electromyography of relevant muscles of the anterior and posterior muscle chain in 16 high-level gymnasts during intentional accelerating movement phases while performing 18 different gymnastics elements (in various body positions to forward and backward, performed on high bar, parallel bars, rings and trampoline), as well as during non-sport specific pivot movements around the longitudinal axis. The succession patterns to generate the acceleration phases during these movements were described and statistically evaluated based on the onset time difference between the muscles of the corresponding muscle chain. In all the analyzed movement phases, the results clearly support the hypothesized succession pattern from punctum fixum to punctum mobile. This principle was further underlined by the finding that the succession patterns do change their direction running through the body when the rotational axis (punctum fixum) has been changed (e.g., high bar or rings [hands] vs. floor or trampoline [feet]). The findings improve our understanding of intersegmental neuromuscular coordination patterns to generate intentional movements most efficiently. This could help to develop more specific methods to facilitate such patterns in particular contexts, thus allowing for shorter motor learning procedures of context-specific key movement sequences in different disciplines of sports, as well as during non-sport specific movements.

## Introduction

Several previous studies of overhead throwing disciplines, such as javelin, identified efficient torque production “from proximal to distal” as being critical to performance [1–4]. Although many movements in sports require such acceleration of the upper extremities while the lower extremities are fixed at the bottom (e.g., throwing movements), some sports movements have to be generated in a position with the hands fixed (e.g., on high bar in gymnastics), while the segment of the lower extremities has to be accelerated by a whip-like effect in order to realize specific elements efficiently. Thus, our idea was that for an effective acceleration of the legs during such movements on the high bar, the torque production has to run inversely from the hands to the legs. Based on these considerations, we modified the aforementioned proximal-to-distal idea, which current “core-stability concepts” are also related to, by changing the focus from the *core* (respectively the center of body mass) to the *rotational axis* a person is currently turning around.

The investigation of gymnastics movements in this context is very useful, because on the one hand gymnastics elements are marked by complex and multifaceted movements in different body positions, around various rotational axes, and to different movement directions. On the other hand, the elements have to be performed in a highly standardized, precise, and efficient way [5, 6] in order to realize the skills successfully, and measurements can be made under standardized laboratory conditions without being perturbed by external influences. The investigation of high-performance gymnastics movements therefore serves as an excellent model to investigate general principles of efficient human movement control. Although numerous studies have investigated the biomechanical aspects of various elements in gymnastics [5–13], relatively few have used electromyography (EMG) in gymnasts [7, 14–17]. Aside from our previous work [18–20], no other studies have examined the principles of intersegmental neuromuscular onset succession patterns during fundamental sequences in gymnastics movements.

### The punctum fixum-punctum mobile model and prior studies

**Fig 1:**
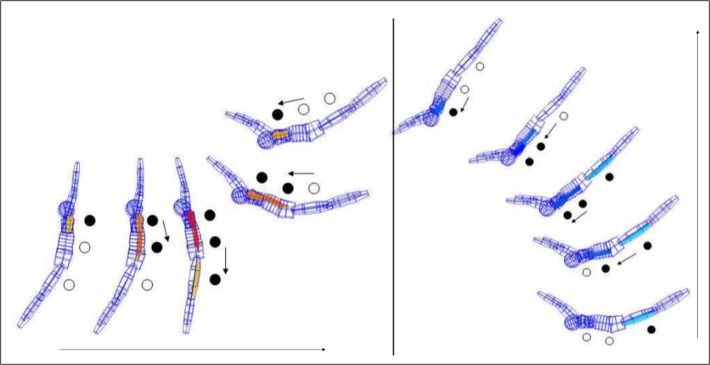
Example of anterior and posterior muscle chain interaction during a giant swing bwd. A lattice model, based on the original kinematic data of a gymnast, is shown with the onsets of the EMG data integrated as colored matrices. During the performance of a giant swing bwd the EMG of the anterior muscle chain (colored in red) was captured from pectoralis major (PM), rectus abdominis (RA) and rectus femoris (RF) and of the posterior muscle chain (colored in blue) from deltoideus, lateral part (DL), erector spinae (ES), and biceps femoris (BF). The direction of neuromuscular onset (and offset) succession is highlighted by black circles. For better demonstration of the overlapping nature of the “spreading” and “recurring” neuromuscular activation, the anterior and posterior muscle chain are each depicted in a separate drawing. For better graphic demonstration and for avoiding overlapping of the positions, the rotational axis is depicted in various positions (the arrows indicate the movement direction). The elected kinematic positions represent each the first frame after the crossing of the onset or offset threshold. See text for more details.

In our prior studies with groups of high level gymnasts we have already described the most efficient strategies of intersegmental neuromuscular onset succession (INOS; see methods for detailled definition) for generation of “whip-like” accelerations, as related to the current *rotational axes* during different movement intentions [18–20]. This concept was introduced as the “punctum fixum–punctum mobile model”. The “punctum fixum” is defined as the part of the body that is fixed at the rotational axis, and the “punctum mobile” is defined as the free part of the body located most distant from the rotational axis. The model includes two main principles: (1) to generate the most effective acceleration of the punctum mobile, the INOS has to run from punctum fixum to punctum mobile; and (2) to generate the most effective transfer of momentum after the punctum mobile has been accelerated, the INOS has to run from punctum mobile back to punctum fixum [19, 20]. These principles are exemplarily depicted in Fig. 1.

According to the terms “punctum fixum” (Pfix) and “punctum mobile” (Pmob), the body segment that is fixed at the rotational axis is defined as “segmentum fixum” (e.g. the arms in a hanging position), the segment that shall be accelerated most efficiently as “segmentum mobile” (e.g. the legs in a hanging position). So, the term “acceleration” within the context of this model refers to whiplike angular acceleration of the segmentum mobile in relation to the segmentum fixum (e.g. legs vs. arms) and therefore is *not* related to accelerations of any body parts in reference to space.

As Fig. 1 demonstrates, the overlapping interaction of the INOS patterns between the agonist and antagonist muscle chain looks like a “wave” of neuromusclular activation running through the body, first “spreading” from Pfix towards Pmob and then “recurring” from Pmob back to Pfix. Therefore we also used the terms “spreading succession” for the succession of the agonist muscle chain from Pfix to Pmob and “recurring succession” for the succeeding deactivation of the agonist muscle chain from Pmob back to Pfix accompanied by the overlapping onset succession from Pmob to Pfix of the antagonist muscle chain.

**Fig 2:**
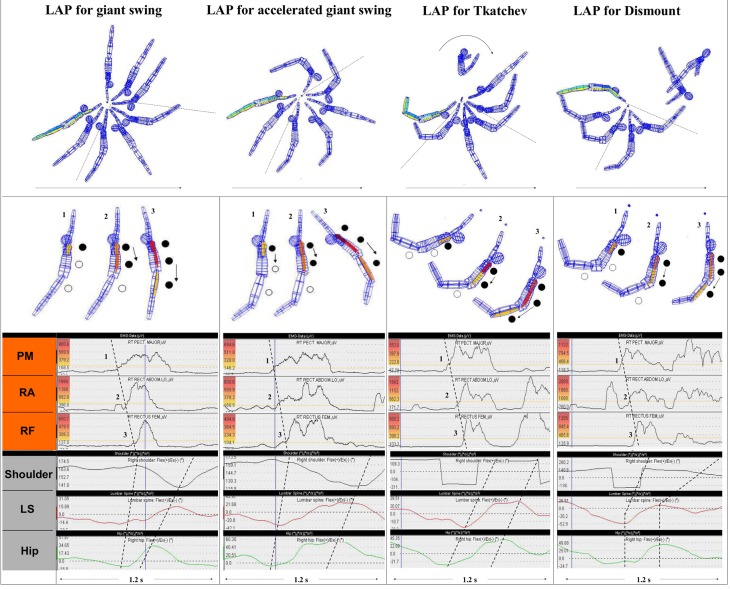
Examples of INOS patterns for generation of whip like leg acceleration phases (LAP) during long hang elements on high bar. The upper row of the figure demonstrates the complete element based on original kinematic data of a gymnast (the arrows indicate the movement direction). The sectors between the dotted lines shall give readers an orientation of the phase of interest (“leg acceleration phase”, each marked by a dynamic change of a body position, slightly curved to backward (“C-minus” position) to a position, slightly curved to forward (“C-plus” position; see methods). The second row in the figure specifically focuses to the INOS patterns for generating the acceleration phase (running from punctum fixum to punctum mobile; highlighted by black circles), with the positions elected of each the first frame after crosssing the onset threshold. The third row shows each the original EMG data of the demonstrated trial, with the onset thresholds (20% treshold line; see methods) connected by dotted lines (the numbers 1, 2 and 3 correlate with the positions as demonstrated in the lattice models above). On each the left side of the EMG plots the color scale is represented, the colored muscle matrices in the segment models of the second row are based on. The colors in the segment models are The last row (at the bottom of the figure) shows the kinematic angle–time characteristics during the movements of the shoulder angle (measured between upper body and upper arms), the angle of pelvis tilt, which is induced by the sagittal movement of the lumbar spine (LS; angle between upper body and pelvis segment) and the hip angle (measured between pelvis segment and thigh). The data is synchronized with EMG based on a frequency of 50Hz. For better demonstration of the intersegmental kinematic succession the integrated dotted lines each connect the onsets (*beginning)* of the angle changes (first dotted line) and the *maximum* of the angles (second dotted line). The fact that these lines run inversely (from the punctum mobile to the punctum fixum) than the dotted lines of the neuromuscular onsets (from the punctum fixum to the punctum mobile) demonstrate that the direction of the intersegmental kinematic succession does not correlate with the direction of the intersegmental neuromuscular succession.

According to principle 1 of the Pfix-Pmob model, we were able to show within series of several long hang elements by gymnasts that in order to generate the most effective whip-like acceleration of the Pmob, the INOS patterns indeed run primarily from the Pfix (hands) to the Pmob (feet) [20]; (see examples of Fig. 2).

As depicted in Fig. 2, the aforementioned study [20] also demonstrated that the kinematic output of the intersegmental movement succession (i.e., succession of closing the leg–trunk angle and shoulder angle) does *not* necessarily correlate with these INOS patterns; rather, it mostly runs inversely (compare EMG data vs. kinematic data in Fig. 2).

Although the kinematic data are, of course, fundamentally influenced by the INOS patterns, there are many additional factors (e.g., the influence of gravity, the varying mass inertia and torque of different body parts during the movements) that further determine the succession of the intersegmental kinematic output of a movement. Therefore, the kinematic output does not allow to derivate sure conclusions regarding to the neuromuscular input, being necessary to realize the desired kinematic output. Teaching methods, being solely based on kinematic analyses could therefore induce wrong or inefficient INOS patterns [20].

**Fig 3:**
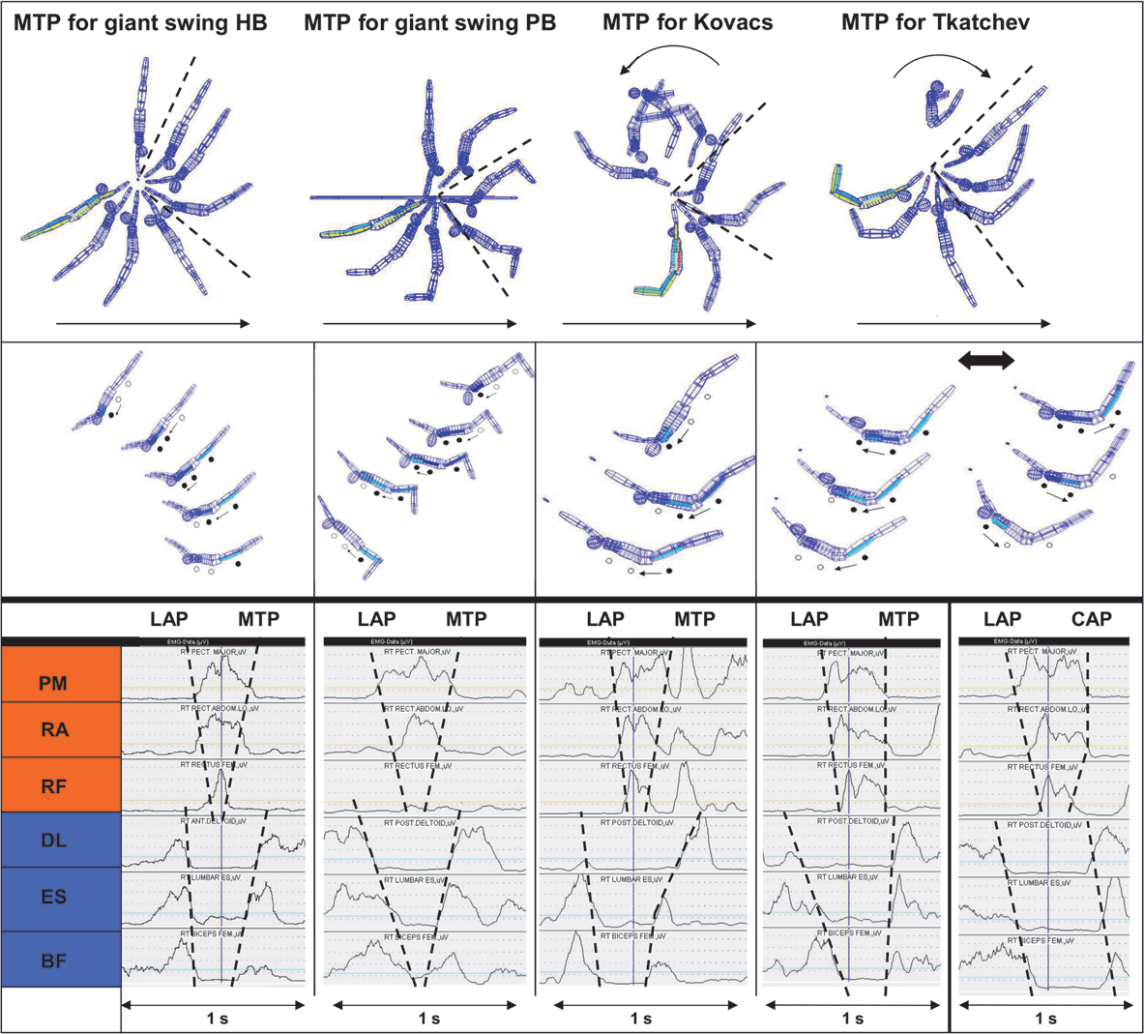
Examples of interactions between the INOS patterns of the anterior and posterior muscle chain during different long hang elements. In the two upper rows the figure demonstrates typical INOS patterns of momentum transfer phases (MTP) during different elements on high bar (HB) and parallel bars (PB)—and additionally, in the lower two rows of the figure it gives an overview about the typical interactions between the anterior and posterior muscle chain during the acceleration phase that is directly followed by the momentum transfer phase, as depicted above. The upper row of the figure shows the complete element based on original kinematic data of a gymnast (the arrows indicate the movement direction). The sectors between the dotted lines shall give readers an orientation of the phase of interest (momentum transfer phase, each marked by a dynamic change of from “C-plus” position (body position slightly curved to forward) to “C-minus” position (slightly curved to backward; see methods). The second row in the figure specifically focuses to the INOS patterns of the MTP (running back from punctum mobile to punctum fixum; highlighted by black circles), with the positions elected of each the first frame after crosssing the onset threshold (see methods). The third and last rows (EMG plots) show each the original synchronized EMG data of the demonstrated trials, with the onset and offset thresholds of the anterior (third row) and posterior (fourth row) muscle chain during the movement (LAP: Leg acceleration phase; MTP: Momentum transfer phase; CAP: Counter-acceleration phase). The dotted lines in the EMG plots connect the crossing points of the EMG signal with the 20% threshold for the onset and offset detection, generating a “double V” constellation that is typical in movements with accceleration phases followed by a transfer of momentum phase. This demonstrates the typical overlapping characteristics of “spreading and “recurring” patterns of the neuromuscular activation (compare also Fig. 1). Only the “Tkatchev 2” shows a deviation of this “double V” constellation (see text for more details).

The results of a further study [19] highlighted principle 2, and they described the intersegmental interactions of the neuromuscular onsets and offsets within and between the anterior and posterior muscle chain (see examples and definitions in Fig. 3). These patterns were regularly marked by the following characteristics: (1) During the activation of a muscle of the anterior muscle chain (AMC) its antagonist of the posterior muscle chain (PMC) is regularly de-activated. This includes that the offsets of the muscles of the agonist chain correspond each with the onsets of the antagonist chain, and vice versa. (2) The phases of intersegmental neuromuscular ‘‘spreading” and the intersegmental neuromuscular ‘‘recurrence” are overlapping each another. That means that the agonists are still ‘‘recurring” while the antagonists already begin to ‘‘spread”, and vice versa (see EMG plots of Fig. 3; compare also Fig. 1).

In all the elements these typical interaction patterns could be identified as to be significantly most frequent. However, during the “Tkatchev element” (the only captured element that includes a change of the rotational direction; Fig. 3), two different INOS patterns of the posterior muscle chain were observed: Technique 1 with a succession from Pmob back to Pfix (as typically identified for the “momentum transfer phase”) and technique 2 with a neuromuscular succession from Pfix to Pmob. The first technique was discussed to more support the transfer of momentum, the second technique to rather support an efficient counter-rotation of the body, induced by a “counter-acceleration” of the antagonist muscle chain towards the Pmob. Both of these components (“momentum transfer” for reaching the hight over the bar and “counter-accleleration” for inducing a more efficient counter-rotation) seem to be necessary requirements to realize the movement [19]. Based on these findings we concluded that there are different INOS patterns concerning the phase *following* the “leg acceleration phase”, depending on the gymnasts intention and the requirements of the specific element (e.g. higher priority to generate an efficient “momentum transfer” component or to generate a more efficient “counter-acceleration” component).

Independent of these intentional strategies of the phase that *follows* the leg acceleration phase, however, in *all* measured elements the INOS from Pfix to Pmob (according to principle 1) for *generating the prior acceleration of the Pmob* was significant. Therefore, in this present study we will further focus to that phase (principle 1) and investigate whether this principle indeed can be generalized as a fundamental prerequisit of neuromuscular coordination for most efficient movement generation.

### Aim of the present study

All the abovementioned measurements [19, 20] were exclusively based on different long hang elements in gymnastics that were performed in the sagittal plane with a leg acceleration phase to forward. To further investigate whether the Pfix–Pmob model is indeed a *fundamental principle* for efficient movement generation in general, the aim of the present study was to examine whether the temporal succession of the INOS for generation of efficient acceleration of the Pmob (principle 1) indeed spreads principally from Pfix to Pmob, independent of the kind of movements, the rotational direction or the current rotational axes. This study specifically focuses on the INOS patterns during accelerating movements around various axes, on various apparatuses, in different body positions, and to different movement directions, as well as during non-sport specific movements (pivot movements). In addition to elucidating general aspects of basic movement sciences, a deeper knowledge of such principles could be useful in the development of more context-specific methods for facilitating motor learning procedures.

## Materials and Methods

### Ethics statement

All measurements were conducted according to the principles expressed in the Declaration of Helsinki and were undertaken with the understanding and written informed consent of each subject or their parents, in case of juvenile participants. The study was supported by the German Institute for Sport Science and was approved by a decision of the German Parliament. We were not required to obtain approval from an additional institutional review board for this study. This was confirmed by a written waiver of the German Institute for Sports Science. No research was conducted outside of our country of residence.

### Participants

Sixteen high-level male gymnasts were recruited from the National Training Centre for Artistic Gymnastics in Stuttgart. They were members of different selection levels, classified as A, B, C and D level members. A level gymnasts are members of the German national team, the B level is defined as second-tier gymnasts competing for entry to the A level team, the C level gymnasts are members of the junior national team, and the D level represent the best area or state junior gymnasts. Six participants were members of the A an B level group (age 18–28 years; training 23–28 h/week), three were former C level gymnasts who had not yet reached the B level (age 18–19 years; training 18–28 h/week), and seven participants were members of the D level selection (age 12–17, training 20–22 h/week).

### General design and procedure of the measurements

**Fig 4:**
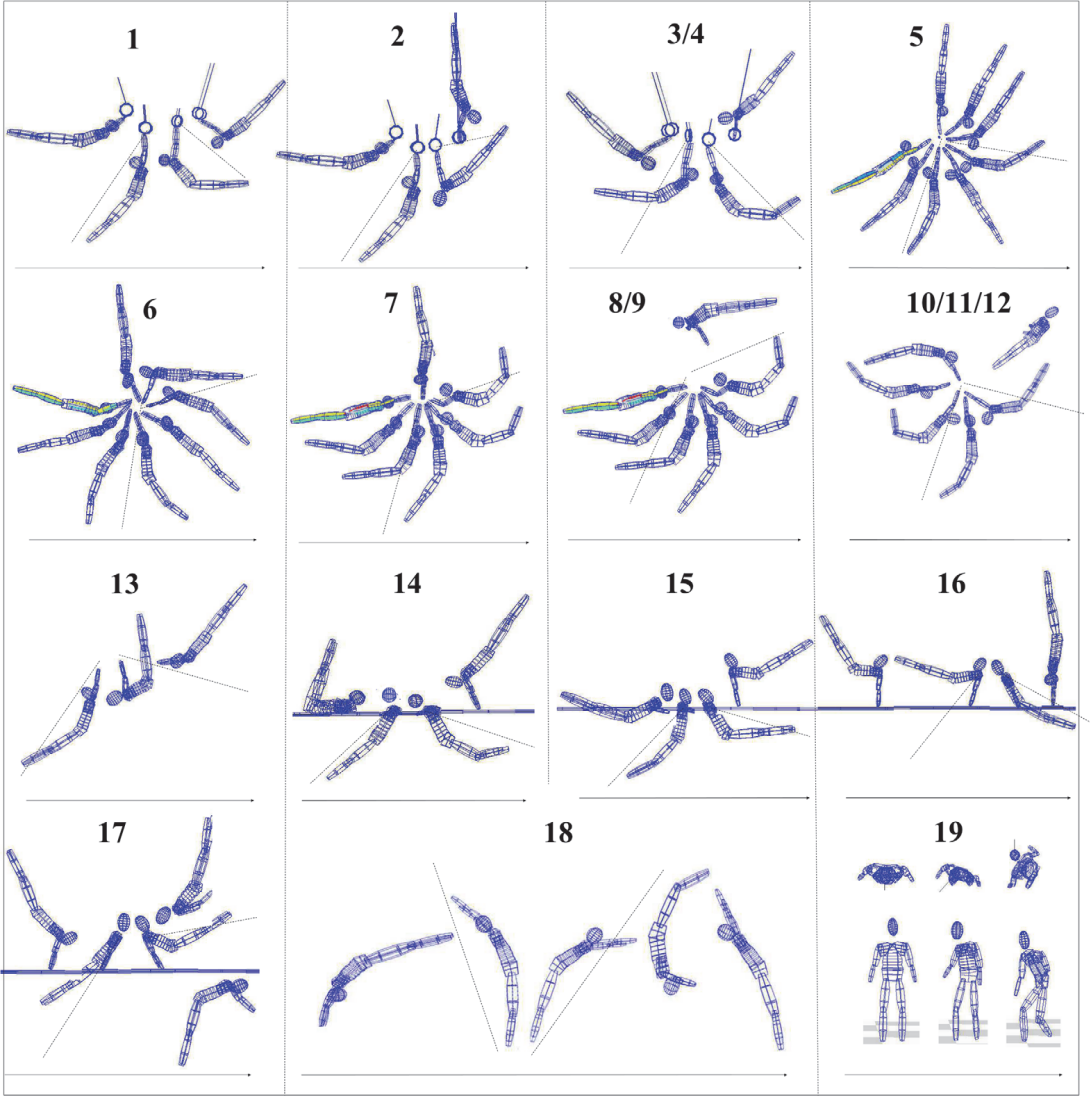
Lattice models of exemplary original kinematic data of all 19 elements. The sectors between the dotted lines of each element approximately represent the phase during which an actively performed acceleration of the punctum mobile takes place. In movement 19, the lower row shows the frontal view, the upper row shows the top perspective.

Wireless surface EMG of defined muscles (see “Technical equipment and preparation”) was measured in the sixteen gymnasts during the demonstration of various movements and elements. They performed a comprehensive bundle of gymnastics elements and complete routines around different axes on several apparatuses in various body positions and movement directions, as well as non-sport specific pivot movements in a standing position (see Fig. 4 and Table 1 for all the movements in detail). The gymnasts were not instructed to pay attention to any special detail during the measurements. The grade of difficulty was determined by the individual level of each gymnast’s abilities. Only those elements that were mastered at a high technical level were performed. Trials that did not meet this standard (e.g., those with heavy failures or that could not be finished successfully) were excluded from further analysis. The essential selection criterion of the elements being analyzed was that they were marked by a clear acceleration intention of the Pmob. For example, the phases of normal “swings to forward (fwd)” during swinging on parallel bars were not included in the analysis, because there is no need to efficiently accelerate within this phase, as long as no element follows. In contrast, before performing a dynamic movement (such as a salto, for dismount) a gymnast has to accelerate efficiently to generate sufficient height and rotation for the dismount. So, these phases were selected for the analysis.

**Fig 5:**
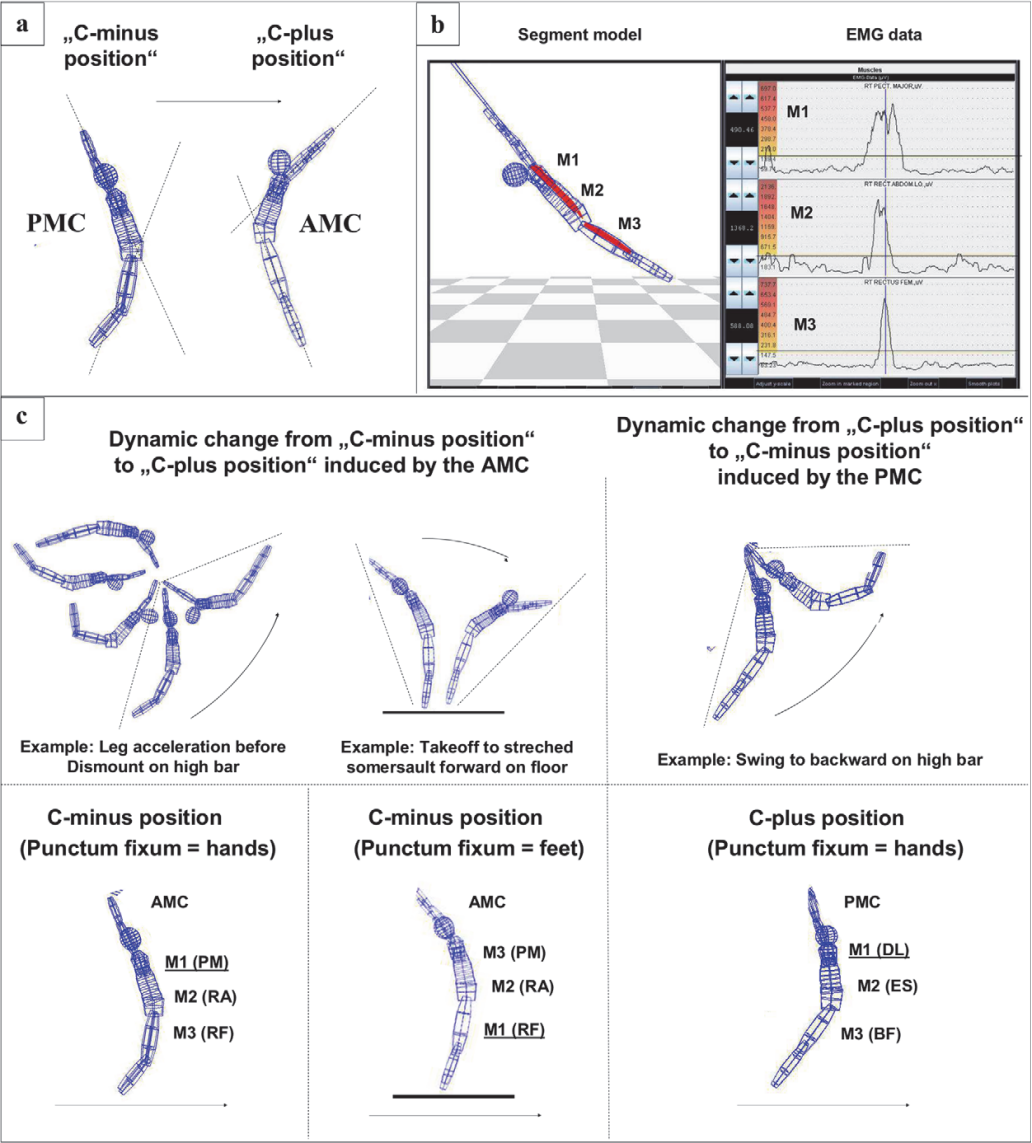
Detection and appellation of the INOS patterns. a) Demonstration of the angles between segmentum fixum and segmentum mobile in the C-minus vs. C-plus position. b) Onset detection: Right: EMG of the anterior muscle chain with m. pectoralis major (PM; M1), m. rectus abdominis (RA; M2) and m. rectus femoris (RF; M3) during a giant swing. The horizontal lines in the plots represent the onset threshold. Their first crossing with the EMG activation is defined as the ‘‘onset time”. On the left side of the EMG plots the color scale is represented, the colored muscle matrices in the segment model are based on. Left: Lattice model of the gymnast (with muscle matrices integrated) at the moment been represented by the vertical line of the EMG on the right. c) Examples of dynamic changes from C-minus to C-plus positions (or vice versa) during different elements around different axes. The appellation of the corresponding muscles depends on the location of the rotational axis (M1 allways next to punctum fixum).

In the sagittal plane such intentional accelerating movements are marked by a whip-like acceleration of the Pmob, which is either directed by a dynamic change of an arched body position (slightly curved backward; we call it here “C minus” position) to a dished body position (more or less curved forward; we call it here “C plus” position) or vice versa (see also “data processing” [Fig. 5] for details). In the horizontal plane (e.g., pivot movements) the whip-like acceleration of the Pmob is marked by a dynamic longitudinal torsion of the Pmob against the Pfix. In total, acceleration phases during 19 movements (*n* = 333) were analyzed (for a more detailed description, see Fig. 1 and Table 1).

**Table 1:** Overview of the analyzed movements.

**Movement group**	**Direction of intersegmental acceleration**	**Element**	**No. in Fig. 1**	***n***
**Long swing elements** on Rings (*n* = 24)	to nose^a^	Swings to fwd	1	8
	to nose	Felge upward to handstand	2	4
	to back	Swings to bwd	3/4	8
	to back	Uprise bwd to support	3/4	4
	to nose	Giant swings bwd^b^	5	30
	to nose	Giant swings fwd	6	32
**Long swing elements** on High bar (*n* = 94)	to back	Back uprise close to handstand	7	11
	to back	Markelov (flight element)	8/9	3
	to back	Voronin (flight element)	8/9	1
	to nose	Dismount: Salto bwd stretched	10/11/12	8
	to nose	Dismount: Double salto bwd stretched with 1/1 turn	10/11/12	7
	to nose	Dismount: Double salto bwd stretched with 2/1 turns	10/11/12	2
**Near axis swings** on High bar (*n* = 34)	to nose	Free hip circle from hanging position	13	34
**Upper arm swings** on Parallel bars (*n* = 15)	to back	Uprise bwd to support	14	11
	to nose	Uprise fwd to support	15	4
**Support swings** on Parallel bars (*n* = 139)	to back	Backswing to handstand	16	111
	to nose	Salto bwd stretched	17	28
**Takeoffs** from Trampoline (*n* = 15)	to nose	Takeoff from handspring fwd to Salto fwd stretched^b^	18	15
**Turns** on Floor (*n* = 12)	to the right^c^	Fast pivot movements in upright standing position	19	12
			Trials in total	333

a: The terms “forward” (fwd) and “backward” (bwd) as used in the elements column refer to the official nomenclature of the Code de Pointage by the International Gymnastics Federation (www.fig-gymnastics.com), which sometimes could be misunderstood in the context of this analysis. Therefore, we described the direction of the intersegmental acceleration by using the terms “to nose” (activation of the anterior muscle chain) and “to back” (activation of the posterior muscle chain) to more clearly define the responsible muscle chain during the acceleration phases.

b: The elements 5 (giant swings bwd on high bar) and 18 (trampoline) were only performed by three participants (age 12–14) in a specific gym with a large trampoline on floor level, whereas the other participants could not be measured on that trampoline. The giant swing bwd was also performed by these three participants to be able to directly compare the INOS patterns between conditions with the rotational axis (punctum fixum) located at the feet (trampoline) vs. conditions with the rotational axis (punctum fixum) located at the hands (giant swings). These participants did not take part in prior studies, so their giant swings bwd were independent data.

c: During the pivot movements, the muscles of the anterior muscle chain were analyzed. Because only the muscles of the right side of the body were captured, only turns to the right could be analyzed.

For a more detailed description of the number of elements and trials per participant see: Table A in S1 Dataset.

### Technical equipment and preparation

#### EMG data

Surface EMG was captured with a wireless eight-channel EMG system (Telemyo 2400T, Noraxon, Scottsdale, AZ, USA). We plotted each the right musculus pectoralis major (PM), musculus rectus abdominis (RA), and musculus rectus femoris (RF) as essential parts of the anterior muscle chain, and the lateral part of musculus deltoideus (DL), which is primarily involved in high forward elevation to open the shoulder angle, musculus erector spinae (ES), and musculus biceps femoris (BF) to represent the posterior muscle chain (see Table 2 for further details). The correct placement of the electrodes over the middle of the muscle belly was achieved by having participants perform isometric contraction in a position as is most common during the elements. For PM, this was a complete forward elevation of the arm trying to close the shoulder angle. For DL the position was identified at 150° forward elevation trying to open the shoulder angle.

**Table 2:** Muscles and functions.

Anterior muscle chain (AMC)		Posterior muscle chain (PMC)	
Synergistic function: Closing the angle between the segmentum fixum and the segmentum mobile towards “C plus” position		Synergistic function: Opening the angle between the segmentum fixum and the segmentum mobile towards “C minus” position	
**Muscle**	**Relevant function**	**Muscle**	**Relevant function**
M. pectoralis major (PM)	Closing the arm-trunk angle towards “C plus” position	M. deltoideus, lateral part (DL)	Opening the arm-trunk angle towards “C minus” position
M. rectus abdominis (RA)	Bending lumbar spine towards “C plus” position; tilt of the pelvis towards “C plus” position (helps to avoid a hyperlordosis of the lumbar spine)	M. erector spinae (ES)	Bending lumbar spine towards “C minus” position; tilt of the pelvis towards “C minus” position (supports hyperlordosis of the lumbar spine)
M. rectus femoris (RF)	Closing the leg-trunk angle towards “C plus” position; (supports knee extension and a deviation towards hyperlordosis of the lumbar spine, if not stabilized)	M. biceps femoris (BF)	Opening the leg-trunk angle towards “C minus” position (supports knee flexion and deviation towards delordosis of the lumbar spine, if not stabilized)

Overview: Muscles of the anterior and posterior muscle chain that were measured by EMG and their most relevant functions (simplified) for achiving the C-positions.

The transmitter unit was affixed using an elastic belt at the lower spine. Another transmitter box was used to receive the trigger signal (for synchronization with the video as described below), which was visualized on a separate channel in the EMG recording. Participants wore an elastic body suit to secure the transmitters, electrodes, and cables in order to not disturb their movements.

The measurements were recorded according to the International Society of Electromyography and Kinesiology’s standards [21]. After shaving and rubbing the skin area with fine sandpaper, a pair of gel-covered AG/AL disc electrodes (radius: 1 cm; Ambu Blue Sensor, Ballerup, Denmark; bandwidth: 0.02–10 kHz) were placed over the muscle belly aligned with the fiber direction, with a distance between electrodes of 2 cm. A reference electrode was attached to the sternum. All electrodes were affixed on the skin with tape to avoid movement artifacts. Electrode skin impedance was accepted at a level of <5 kOhm. The following standards of EMG detection were used, as specified by the manufacturer: input impedance: >100 MOhm; CMRR: >100 dB; and SNR: baseline noise < 1 μV RMS. The raw EMG signals were bandpass filtered (10–500 Hz) and sampled at 1500 Hz, AD-converted (12 bit) and stored for further processing in a personal computer (see data processing).

#### Video data

Additionally, a 50-Hz video camera (shutter: 350 frames/second) was used for visual control of the elements and for documentation. The camera was synchronized with the EMG by a LED, which was placed in the focus of the camera and connected with the trigger signal to ensure a frame-based synchronization of the capturing systems.

Some elements of the video sequences were processed by a 2D videokinemetric system (custom-made software: 2D-Mess, developed by the Institute of Applied Training Science, Leipzig) for a better demonstration and visualization of the movements within a lattice model. Besides generating a lattice model, custom-made software (Vismo; von Laßberg and Reimann [22]) also allowed synchronization and visualization of the neuromuscular activation in the model by depicting the EMG data as colored matrices of the muscles, being represented in the model with the moment of crossing the onset threshold (for more detailed information about the detection of the onset threshold of the EMG data, see Data processing). With regard to the results of [20] the analysis of kinematics will not lead to further relevant information concerning the principles of INOS patterns (compare Introduction). Therefore only a small part of the demonstrated elements was captured by that procedure, to enable exemplary data visualization for clearer graphic demonstration (see Fig. 4).

### Data processing and detection of the INOS patterns

For further analysis the EMG data were full-wave rectified and smoothed over a constant time window of 25 ms by using the software Myoresearch (version 1.06.60, Noraxon, Scottsdale, AZ, USA).

The data analysis for all elements was primarily focused on the intersegmental neuromuscular onset succession (INOS) of the corresponding muscle chain (anterior or posterior) being activated to realize the acceleration phase of the different movements (see Table 1). The INOS was defined as the temporal succession of the EMG onsets concerning the defined muscles of the anterior and posterior muscle chain (see Table 2). The INOS seems to be the best parameter to represent temporal succession for the analysis of general principles of temporal coordination during dynamic movements. This is due to the fact that the onset during dynamic muscular activation is usually based on a sharp and clear slope increase of the EMG signal that can be easily detected by the analysis software, when the slope crosses the onset threshold (see below for further details). Other parameters determined from the EMG data such as *peaks*, *amplitudes or integrals* were not part of this study because they do not provide valid information about the intersegmental timing [23, p. 8] the Pfix–Pmob model is based on and therefore they were not essential for verification of the hypothesis concerning the intersegmental onset succession.

The procedure to identify the INOS patterns was defined as in our prior studies [19, 20]. The patterns begin with the onset time of the first muscle, which is part of the responsible muscle chain which induces the change from “C-minus” to “C-plus” body position, or vice versa (Fig. 5). The exact onset time was defined as the frame of the EMG plot that crosses the onset threshold line. It was only accepted as “onset frame” if it remained over the threshold line until the peak of the signal was reached. The threshold was set at 20% of the maximum amplitude of the measured acceleration sequence. This 20% threshold is often used in onset analyses [23]. In own prior studies with movements in gymnasts, this threshold was further identified as the best compromise between a sufficient filtering of the muscular ground activation without cutting off essential patterns of voluntary activation sequences, and it assured an unambiguous crossing of the threshold line to enable clear identification of the onset time.

The muscles of both the anterior and posterior muscle chains were numbered, with muscle 1 (M1) being nearest to the rotational axis (nearest to Pfix) and muscle 3 (M3) the furthest away (nearest to Pmob). Thus, the INOS pattern from Pfix to Pmob is standardized by the onset sequence M1-M2-M3 (or only: 1-2-3). (Fig. 5C)

The zero point of each measurement was defined as the beginning of M1, independent of whether M1 was really activated first or not. Therefore, the onset of M1 was standardized at a value of 0 s. From this frame onward the times to the onsets of M2 and M3 were measured; they were defined as positive values if the onset began later than M1 and by negative values if activation began prior to M1. Thus, the pure pattern 1-2-3 is marked by positive values of M2 and M3 and with M2 < M3. Negative values of M2 and/or M3 would contradict the onset succession from Pfix to Pmob, as formulated in the hypothesis.

### Statistical analyses

All data were described by calculating the mean and standard deviation (SD) of the onset times, related to M1. Effect sizes were calculated using Cohen’s *d* [*d* = (mean *x*—mean *y*)/mean SD], with values of 0.2 < *d* < 0.5 defined as small effects, 0.5 < *d* < 0.8 as moderate effects, and > 0.8 as large effects [24]. A one-way ANOVA was conducted for the factor of the differences between the onset times of muscles M1, M2, and M3. In case of significant values, a Bonferroni post hoc calculation of multiple testing was performed with pairwise comparisons. It was confirmed that the data was normally distributed (i.e. using Kolmogorov-Smirnov). Homogeneity of variance was controlled (Levene test) and the post-hoc calculation accordingly adjusted in case of failures (i.e. using Games Howell). The alpha level was set at α = 0.05. SPSS 20 (IBM, Armonk, NY, USA) was used to conduct the statistical analyses.

Because of the specific post hoc design of this study, the individual counts of trials per element were not completely balanced. This can slightly bias the values of the ANOVA. One way to adapt to such imbalance is to elect specific trials to generate equal numbers of trials per participant and element. By such a reduction procedure, however, the total number of trials would have been essentially reduced and data transparency would have been affected. Therefore, in this study all trails that were performed (excepted failed trials) were included in the statistical analysis.

To prevent misinterpretations of the pure ANOVA results, additional descriptive analyses of the individual counts of trials and the proportional representation of each gymnast’s INOS patterns (in %) are offered. For this, the individual succession of the defined muscles was counted by inspection of each trial as a pure *order* of the muscle onset succession. For example, the pattern 1-2-3 represents the succession M1-M2-M3, *independent* of the time differences between the musles (in contrast to the ANOVA approach). Within this descriptive analysis, latencies between muscle onsets shorter than 0.01 s were defined as “simultaneous”. This takes into account that the delay between the actual onset of myoelectrical activity and the onset of recorded EMG can vary, due to the current position of the electrodes relative to the innervation zone and its spatial variabilty caused by differences of muscle length during dynamic movements. For example, a location of the electrodes 4 cm from the innervation zone is described to result in a delay up to 0.01 s [23].

## Results

### Results of the ANOVA

**Fig 6:**
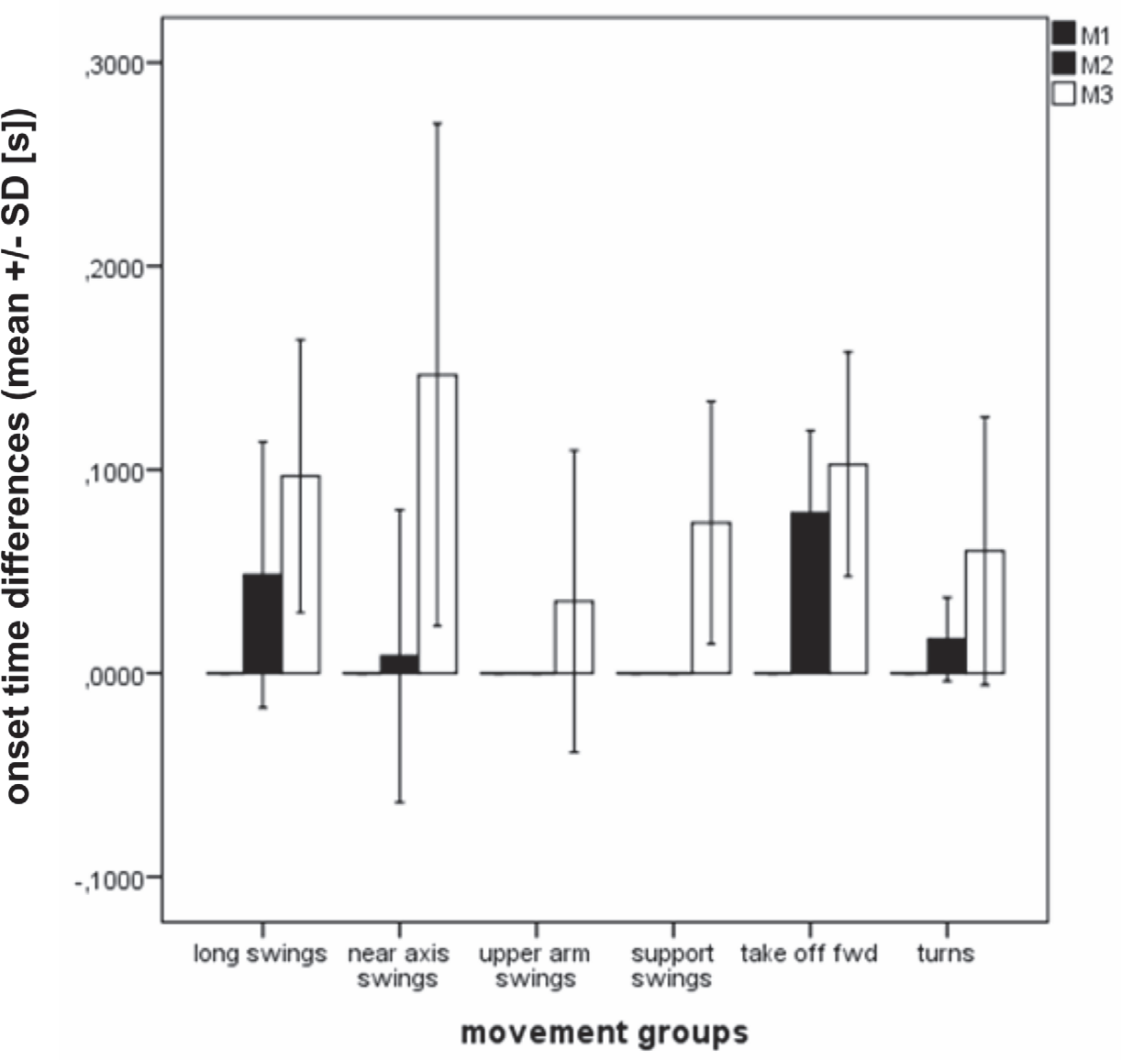
Mean ± standard deviations within the different movement groups. The data refer to the onset times related to muscle 1 (M1) for generating the acceleration phases, according to the six groups as defined in Table 1.

Fig. 6 shows the onset time differences between M1, M2, and M3, calculated for each of the six general movement groups, as defined in Table 1.

The ANOVA over all the movement groups together was highly significant (F = 261.1; p < 0.001).

Also the ANOVA calculated for each of the six movement groups were significant (Long swings (LS): F = 92.99; p < 0.001; Near axis swing (NAS): F = 33.86; p < 0.001; Upper arm swings (UAS): F = 3.40; p = 0.043; Support swings (SupS): F = 234.96; p < 0.001; Take off during handspring forward-Salto streched (HSS): F = 27.67; p < 0.001; Turns: F = 7.24; p = 0.002).

The post hoc analyses concerning the time differences between M1 and M3 were also significant. LS (p < 0.001; d = 2.90), NAS (p < 0.001; d = 2.37), HSS (p < 0.001; d = 3.75) and Turns (p = 0. 023; d = 1.82). In UAS and SupS the shoulder axis is the rotational axis itself. Therefore M1 was not measured and post hoc analyses were not calculated. However, the post hoc analyses between M1 and M2, and also between M2 and M3, as well, were not allways significant (see Table B in S1 Dataset) for detailed values).

**Fig 7:**
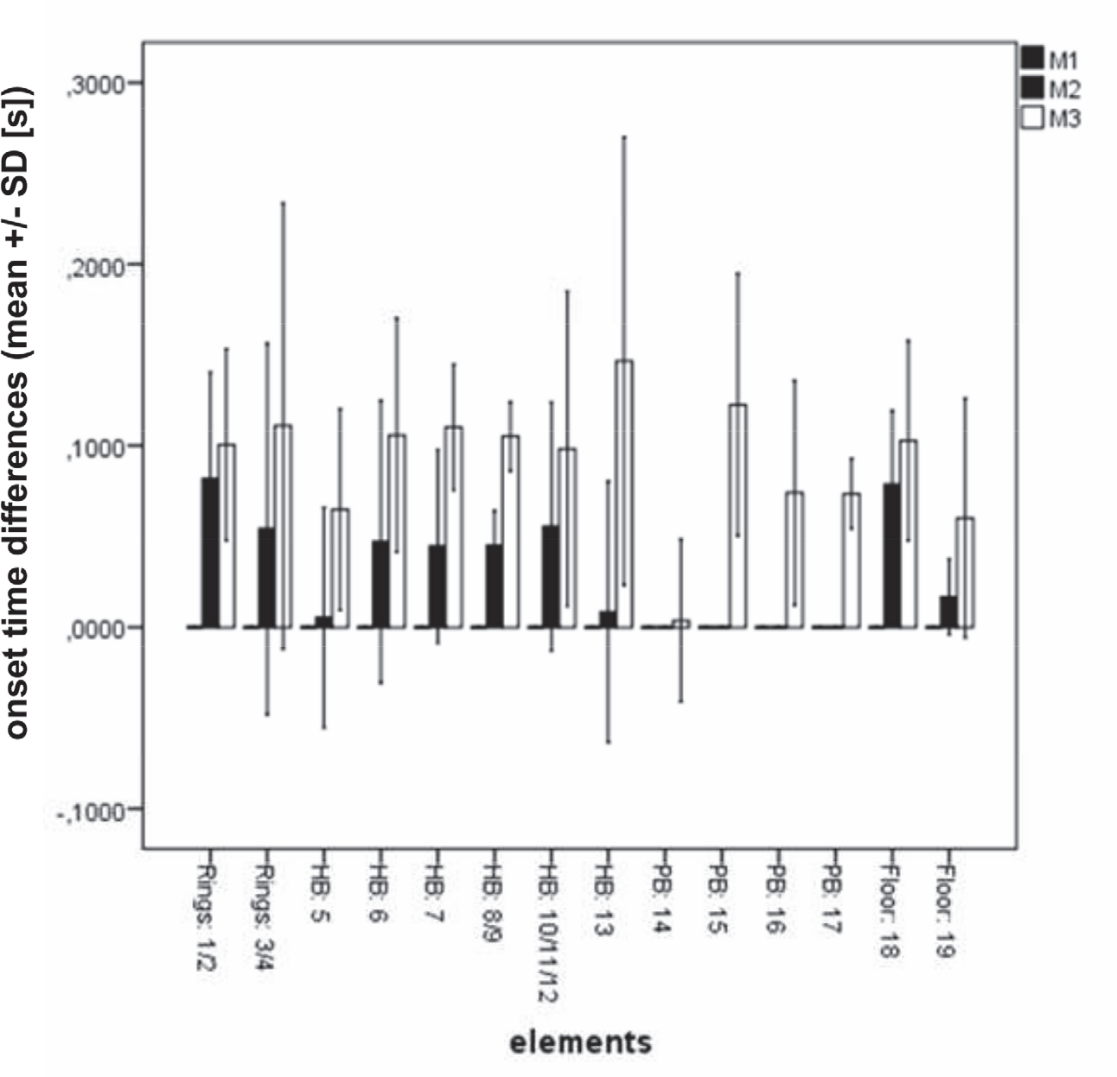
Mean ± standard deviations within the different elements. The data refer to the onset times related to the muscle 1 (M1) for generating the acceleration phases, according to the element numbers as defined in Fig. 1. HB: high bar; PB: parallel bars. For better readability, some of the elements were combined into one bar in the figure.

The analysis, subdivided into the singular movements is depicted in Fig. 7.

With a single exception of one element (element 14 [UAS]) the ANOVA results of all measured elements were significant. Also the post hoc analyses between M1 to M3 were significant in these elements. However, the post hoc analyses M1 to M2, and M2 to M3, as well, were not allways significant (see Table C in S1 Dataset) for all data).

### Descriptive analysis of individual INOS patterns

Table 3 gives a more detailed insight into the individual representation of each gymnast’s patterns distribution concerning the six movement groups and lists the percentages of INOS patterns over all elements in total.

**Table 3:** Descriptive analysis of the percent of individual counts of demonstrated patterns within the six element groups per subject.

	**Percent of individual counts of demonstrated patterns (patterns without any counts are not listed)**																
**Subjects**	P1	P2	P3	P4	P5	P6	P7	P8	P9	P10	P11	P12	P13	P14	P15	P16	
**Patterns** *	**Long swings, summarized all (LS: *n* = 103)**																**% in total**
**1-2-3**	85.7	92.9	100	100	100	83.3	100	33.3	33.3		100		43.7	80	90	100	76.1
**2-1-3**		7.1				16.7			50	100			25				13.3
**1-3-2**	14.3												25		10		3.3
**2-3-1**								66.7	16.7				6.3	20			7.3
**Sum**	100	100	100	100	100	100	100	100	100	100	100	100	100	100	100	100	100
	**Near axis swings, summarized all (NAS: *n* = 31)**																**% in total**
**1-2-3**	80		33.3						100				33.3				30.8
**2-1-3**	20		66.7			50	100	100			66.7		66.7				58.8
**1-3-2**						25					33.3						7.3
**2-3-1**						25											3.1
**Sum**	100		100			100	100	100	100		100		100				100
	**Upper arm swings, summarized all (UAS: *n* = 15)**																**% in total**
**2–3** **					100	100	100		100								80
**3–2**	100																20
**Sum**	100				100	100	100		100								100
	**Support swings, summarized all (SupS: *n* = 138)**																**% in total**
**2–3** **	100				100	90.6	100	100	100	100			100				98.8
**3–2**						9.4											1.2
**Sum**	100				100	100	100	100	100	100			100				100
	**Handspring-Salto on Trampoline, summarized all (HSS: *n* = 15)**																**% in total**
**1-2-3**														80	80	100	86.7
**1-3-2**														20	20		13.3
**Sum**														100	100	100	100
	**Pivot movements, summarized all (Turns: *n* = 9)**																**% in total**
**1-2-3**					100	100	100	50	100	100		100					81.3
**3-1-2**				100													12.5
**2-3-1**								50									6.2
**Sum**				100	100	100	100	100	100	100		100					100
**All elements**	**All elements summarized (patterns in % of counts) without UAS and SupS** **																**% in total**
**1-2-3**	82.9	92.9	66.7	50	100	61.2	66.7	27.8	77.8	50	50	100	38.6	80	85	100	70.6
**2-1-3**	10	7.1	33.3			22.2	33.3	33.3	16.7	50	33.3		45.8		15		18.8
**1-3-2**	7.1					8.3					16.7		12.5	10			3.4
**3-1-2**				50									3.1				3.3
**2-3-1**						8.3		38.9	5.5					10			3.9
**3-2-1**																	0
**Sum**	100	100	100	100	100	100	100	100	100	100	100	100	100	100	100	100	100
	**UAS and SupS** ** **summarized (patterns in % of counts)**																**% in total**
**2–3** **	50				100	92.7	100	100	100	100			100				92.8
**3–2**	50					7.3											7.2
	100				100	100	100	100	100	100			100				100
**Subjects**	P1	P2	P3	P4	P5	P6	P7	P8	P9	P10	P11	P12	P13	P14	P15	P16	

In the left column the patterns are listed, as demonstrated during the performance of the elements within the six element groups. The patterns are sorted by the pattern 1-2-3 in the first row (2–3 in upper arm swings and support swings**). The patterns in the other lines of each element group are sorted by the criterion of being closest to the pure pattern 1-2-3 running from punctum fixum to punctum mobile*. However, patterns that never appeared are not listed within the elements groups.

* The complete ranking of the possible patterns being most similar to 1-2-3 is: 1-2-3, 2-1-3 (both ending with M3); 1-3-2, 3-1-2 (both ending with M2); 2-3-1, 3-2-1 (both ending with M1).

** In upper arm swings (UAS) and support swings (SupS), the shoulder axis is the rotational axis itself and the shoulder muscles have to stabilize this axis during these movements. Therefore, in these two movement groups the shoulder muscles were not included in the analysis, and so only M2 and M3 were analyzed by their succession patterns.

Twenty-two of the measured 333 trials could not be clearly matched to a specific pattern because of simultaneous onsets (time diferences < 0.01 s) of at least two muscles (see methods). Therefore, Table 3 refers to a maximum of 311 trails, in total.

To provide further details of the data listed in these tables, an additional synopsis of all the patterns counts and percentages for each of the element demonstrated by each participant is attached in the Supporting Information (see Table D in S1 Dataset).

The data in Table 3 show, that the pattern 1-2-3 is clearly preferred, followed by the pattern 2-1-3. Both patterns represent M3 as the last muscle, and so both patterns generally underline the hypothesis that the Pmob is activated last. Beside the percentages as listed in Table 3 the following percentages over *all elements together* (UAS and SupS summarized) can be calculated: Among the 311 analyzed trials, 111 showed the pattern 1-2-3 and 145 trials (in UAS and SupS) the patterns 2–3, both representing the onset succession from Pfix to Pmob (see S1 Dataset). In total, these patterns represented 82.32% of the 311 trials. Another 28 trials showed the pattern 2-1-3. Therefore, at least 91.32% of the 311 trials were marked by patterns with the Pmob being activated last. Other patterns were observed with the following proportions: 2-3-1 (2.57%), 1-3-2 (3.22%), 3-1-2 (0.32%), and 3–2 (UAS and SupS: 2.57%).

Not a single trial showed the pattern 3-2-1, which would represent the pure succession pattern from Pmob to Pfix. This distribution shows that the patterns are clearly weighted, preferring the succession patterns running from Pfix to Pmob, as hypothesized.

Looking specifically at the elements with Pfix at the feet (HSS and turns, *n* = 24 in total), 20 trials were marked by the pattern 1-2-3 (83.33%), and the remaining four trails showed the pattern 1-3-2 (8.33%), 3-1-2 (4.17%), and 2-3-1 (4.17%). From the 11 participants that performed one of these elements (HSS or turns) 10 subjects showed the patterns 1-2-3 (running inversely from feet to hands). Intra-individual comparisons of the gymnasts patterns between elements with Pfix at the feet vs. elements with Pfix at the hands demonstrate that 8 of these 11 gymnasts showed a complete reversal of their patterns 1-2-3 (running from feet to hands in elements with Pfix at the feet vs. running from hands to feet in elements with Pfix at the hands) in 18 of the 23 direct intra-individual comparisons (compare Table D in S1 Dataset).

**Fig 8:**
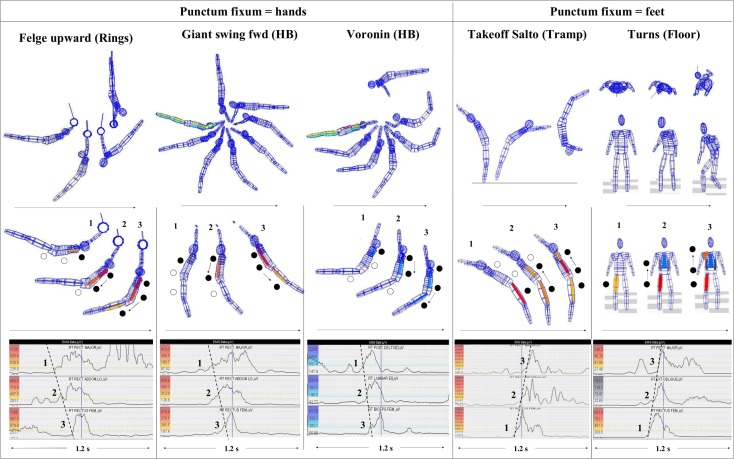
Examples of original data gathered during some movements of the present study. The top row shows the complete element based on original kinematic data of a gymnast (arrows indicate movement direction). The second row shows the INOS patterns for generating the acceleration phases (from punctum fixum to punctum mobile), with the positions selected of the first frame after crossing the onset threshold (see methods). On each the left side of the EMG plots the color scales are represented, the colored muscle matrizes in the segment models of the second row are based on. The bottom row shows the original EMG data of the demonstrated trial. The dotted lines in the EMG plots connect the crossing points of the EMG signal with the 20% threshold line to better clarify the patterns of the intersegmental neuromuscular onset succession (1, 2, and 3 correlate with the positions in the lattice models above). Upper plot: upper extremities (PM/DL); middle plot: mid body (RA/ES); additionally, for a better depiction in the frontal view of the presented pivot movement (floor), also the left and right musculus obliqui externi were captured in that example; lower plot: lower extremities (RF/BF). Depending on the location of the rotational axis (punctum fixum) the onsets run from the hands to the feet (during elements with punctum fixum = hands) or from the feet to the hands (during elements with punctum fixum = feet).

Summarized, Fig. 8 exemplarily outlines some of the present results by showing typical EMG patterns during some of the movements around different axes, in different body positions, and during different movement directions.

## Discussion

### General interpretation of the results

According to ANOVA, the mean values of the onset succession across all elements and during each of the six movement groups clearly underline the trend towards the onset succession from Pfix (M1) to Pmob (M3). The time delays between the onset of M1, M2, and M3 of these patterns across all elements, in total, were highly significant. In each of the six movement groups, they were significant or highly significant in most cases (see also Tables B and C in S1 Dataset). Furthermore, because these patterns of neuromuscular onsets show very short ranges of maximum time lags (between 0.01 and 0.2 s, which includes the measurement error), this highlights the participants’ ability to achieve a high grade of intersegmental temporal differentiation and coordinative control. Although even high-level gymnasts never represent *absolute* perfection, we can assume that their main patterns generally represent an effective way to realize the captured movements. Also the descriptive analyses of the individual patterns clearly highlight these results (Table 3). If the INOS patterns were generated randomly or by measurement errors, one might expect that all six possible combinations of the succession patterns (1-2-3, 1-3-2, 2-1-3, 2-3-1, 3-1-2, and 3-2-1) would be represented by about 16.67% of all the measurements. The results, however, indicate that the distribution of the patterns is not balanced but rather weighted toward patterns that represent INOS pattern running from Pfix to Pmob. So, both statistical approaches (ANOVA and proportional INOS analysis) clearly underline the principle 1 of the Pfix-Pmob model, as hypothesized.

Together with our previous studies [19, 20], acceleration phases of 25 different movements (with more than 450 analyzed trials, in total) were measured in high-performance athletes. All the movement sequences analyzed in these studies referred to the same muscles as in the present study and did significantly support the proposed hypothesis of the model. Likewise, the findings of Frère and Hug [7] seem to support these results during the performance of giant swings backward in a group of high-level gymnasts. Although it was not the aim of their study to measure INOS, their EMG data of PM, RA, and RF (as presented in their Fig. 3) also seem to illustrate this onset succession from PM (first) to RF (last), in accordance to our findings.

From a more biomechanical point of view, the Pfix-Pmob concept could be debated by the argument that the measurement of singular muscles within a muscle chain is an excessive simplification of the real interactions between synergistic and antagonistic functions of different muscles between the anterior and posterior muscle chain and within these muscle chains. It is also the case that we did not clearly describe how a “muscle chain” is defined exactly, because the relevant synergistic muscles within a functional muscle chain can differ fundamentally depending on the kind of movement, its intention, and the plane or rotational axis of a specific movement. However, this is not our question. The focus of the Pfix–Pmob model relies exclusively on the temporal succession of the included muscles in relation to the distance from the rotational axis.

Even though all these results clearly support the INOS patterns from Pfix to Pmob, a further objection could be that these patterns are not voluntarily coordinated but rather determined by other factors. So, it could be argued that the specific succession could be explained by neural factors (e.g., different anatomical latencies due to nerve conduction, caused by differences in the length of the neural path between the muscles that were measured). Another argument against a voluntary succession could be that the neuromuscular onsets are caused by reflectory mechanisms (e.g., myostatic reflexes). Of course, it cannot be excluded that both of these factors could play a role during the movements.

However, there are several counter-arguments as to why these factors do not affect the results significantly. First, if anatomical factors cause the INOS patterns, it cannot be explained why the INOS successions change direction depending on the current rotational axis, as demonstrated by the comparison of giants swings (Pfix = hands) vs. takeoffs from the trampoline (Pfix = feet). To give better evidence for this phenomenon of context-specific intraindividual changes of the succession direction, we recruited three independent participants who performed the two elements in an extra session (see legend of Table 1). Further, the INOS patterns also change direction depending on different movement *intentions*. For example, concerning the posterior muscle chain, the results showed an onset activation from Pfix to Pmob to generate an *acceleration* of Pmob “to the back” (e.g., during the elements 3, 4, 7, 8, 9, 14, 16). However, the onsets run inversely (from Pmob to Pfix) to generate a *transfer of momentum* after prior acceleration of Pmob “to the nose” (e.g., during giant swings backward on high bar or parallel bars, as demonstrated in Fig. 3). Second, whereas the results show the leading INOS pattern 1-2-3, there were also inter- and intraindividual trials with deviations from this pattern. These findings also indicate a more coordinative background of the patterns than for reflectory or even anatomical reasons, because in the latter case all the patterns should be equal. Third, the assumption that the results could be caused by stretch reflex mechanisms, for example, also is not convincing, because the patterns appeared not only during dynamic movements (with a phase of prior muscle extension), but also during the pivots, which were started from a quiet upright standing position.

**Fig 9:**
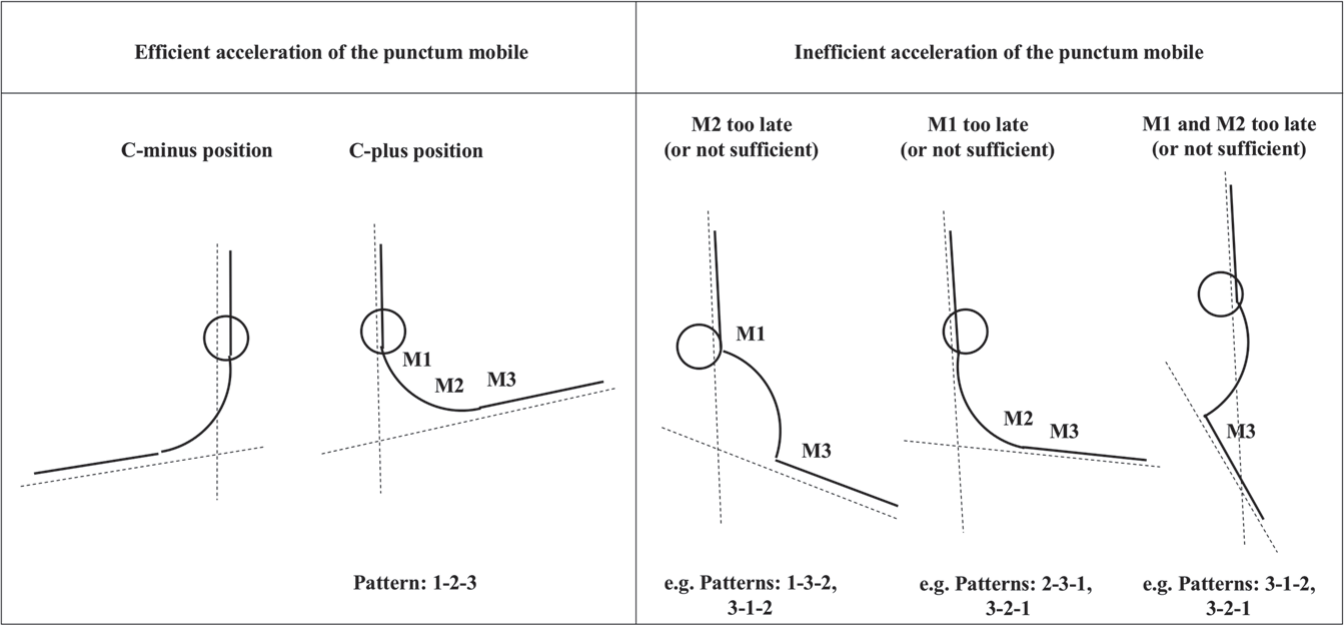
Efficient vs. inefficient INOS patterns. On the left side of the figure, the angles between the segmentum fixum and segmentum mobile (C-minus vs. C-plus position) are schematically depicted during an efficient INOS activation from punctum fixum to punctum mobile (pattern 1-2-3). On the right side several examples of inefficient INOS patterns are demonstrated. All these patterns result in a diminished angle between segmentum fixum and segmentum mobile. Additionally, the drawings demonstrate the resulting deviations of body segments, caused by deviating INOS patterns, as listed in the bottom line.

The abovementioned considerations lead to the question, what functional mechanisms in detail cause the described “principle 1” within the Pfix—Pmob model. As pointed out earlier [18] we postulate that it is necessary to begin the neuromuscular activation from the Pfix, running to the Pmob to effectively compensate for the inertial latencies of the body segments (also with regard to their contractile and elastic properties), which are larger the closer they are to the Pfix. In case that the neuromuscular succession counteracts to that principle, the multisegmental accelerations will partly neutralize each another and lose efficiency concerning the resulting angular acceleration between segmentum fixum and segmentum mobile, as demonstrated in Fig. 9. The figure makes also clear that beside the loss of efficiency such patterns can also result in higher loads to passive structures of the musculo-skeletal system such as joints, ligaments and connective tissues (e.g. to vertebral structures by a resulting hyperlodosis).

An aspect the EMG data is *not* able to measure is which specific kind of muscular function takes place during the neuromuscular activation phases. So, it is not possible to distinguish between eccentric, isometric or concentric muscular activation.

This point is tightly associated with questions of the intrinsic muscular properties and their specific architecture of contractile and elastic components. It must be assumed that during such dynamic counteracting movements on a high performance level, as it was performed in the current study, there is a well coordinated interaction between the contractile elements of the muscle fibers and the specific characteristics of elastic structures (aponeuroses, tendons, connective tissue) within a muscle-tendon unit. This effect can efficiently help to reduce the extent of eccentric-concentric muscular work and save energy during movements, in case it is well-coordinated [25]. Further, it can not be excluded that also mechanisms like succeeding strech-shortening cycles and interactions of contractile and elastic structures *between* the muscles of the active muscle chain could play an additional role, which could functionally enhance the intersegmental “whip like effect” that supports a most efficient acceleration of the Pmob. Such succeeding strech-shortening cycles could be based by streching of each the *following* muscle that will be activated within the 1-2-3 pattern, induced by the prior contraction of the muscle that is each activated *before*. For example, the initial contraction of M1 during the extended C-minus position in the beginning of a 1-2-3 INOS pattern could lead to a slightly further extension of M2, which itself will consecutively enhance its “preload” prior to its own contraction. The following contraction of M2 again leads to an even further extension of M3 that, again, could therefore enhance its preload and its final whip like contraction. In particular, such effects could be relevant during elements with such extraordinary dynamic switches between C-plus and C-minus positions in high performance gymnastics elements, even more supported by the overlapping nature of the INOS interaction between the anterior and posterior muscle chain (Fig. 3). In this context, also the interaction of all these aspects with further *external* elastic properties of the apparatuses (e.g the high bar) might be a relevant factor which supports movements efficiency within this complete “man/apparatus interaction system” [5, 6]. However, the specific roles and the functional relevance of such mechanisms within the Pfix—Pmob model can not be judged by the approach of the present study.

### Further aspects and implications

A noteworthy finding is that the pattern 1-2-3 was not only related to movements with an acceleration of the Pmob in the same direction as the body rotates. This pattern was also seen in the case of accelerations against the body’s rotational direction to generate a shortening of the pendulum system by bringing the center of gravity closer to the rotational axis. For example, in Fig. 8 compare Felge upward (rings) vs. giant swings forward (HB). These elements are marked by an inversed rotational direction, but both driven by a leg acceleration “*toward the nose*”, achieved by the *anterior muscle chain*; in contrast to this, in the case of Voronin (HB), it is the same rotational direction as giant swing forward, but with leg acceleration “*to the back*”, achieved by the *posterior muscle chain*.

Although most movements measured in the present study were conducted in the *sagittal* plane, the pivot movements also clearly supported the Pfix to Pmob principle in the *horizontal* plane (Fig. 8). Because the Pfix in this case was also changed (feet instead of hands, as during the HSS), the neuromuscular succession also runs inversely, from the legs toward the upper extremities. The results during longitudinal turns also support the hypothesis, indicating that these principles refer to any axis a person turns around. In addition, one of our previous studies [18] indicated that the Pfix–Pmob principle not only applies to pure spinal-motor patterns but also includes eye movement coordination, with the eyes being defined as the Pmob during fast intentional reorienting body turns. These results demonstrated that the eyes do *not* turn first to be redirected to the new target most effectively (as one might expect because of their lowest mass inertia), but rather they are activated as the *last* segment, according to the aforementioned principle (see Video 5 in [18]). Such fast redirecting eye movements (known as saccades) cause blurring of the visual image. Therefore it needs to be as quickly and accurately as possible to minimise loss of vision. So, the main demand for saccadic eye movements is “efficiency”. As discussed in [18], the succession from Pfix to Pmob enables a more synchronized summing up of the intersegmental accelerations of body segments (up to the eyes as the Pmob in that case) for generating the most effective gaze shift to a new target with minimized loss of vision. Similar findings that generally support these results were reported by Bizzi [26] and Anastasopoulos et al. [27]. These findings further highlight the general meaning of the Pfix—Pmob model also concerning more sensory integrative aspects of human eye-head-body interactions and intersegmental motor control. They open the discussion as to whether general mechanisms of both intersegmental spinalmotor and also cervico-oculomotor control, as well, could be based on the same functional principles [18]. If this is the case, it could be worthwhile to more specifically look for such interdisciplinary parallels to get a more integrative understanding of interactions between perception, cognition and action, concerning general questions of sensory integrative coordination and human movement control.

To further complete the Pfix-Pmob model, future studies could be related to INOS patterns during planned vs. reactive (non-predictive) scenarios of intersegmental accelerations (compare [18] and [26]) and to the question whether the model can be also extended to more linear directed movements (induced by counteracting intersegmental rotations).

In summary, the findings of this study indicate that the INOS from Pfix to Pmob is a general principle underlying an efficient movement acceleration of body segments, according to “principle 1” of the Pfix-Pmob model. This knowledge could help to enhance effective motor learning by an earlier optimization and facilitation of these patterns and to avoid long and ineffective detours during the learning process, which moreover can cause unnecessary loads to musculoskeletal structures. Future studies are in preparation to investigate which kinds of methods would be best for facilitating these coordination patterns in practical work to perform movement sequences more efficiently.

## Supporting Information

S1 Dataset Table A. All elements and trials per subject analyzed within the ANOVA. **Table B**. ANOVA within the six element groups. F and p values of ANOVA; post hoc analysis in case of significant values: Mean values, standard deviations (SD), Cohen’s *d*, and *p*-values for the temporal differences (T) of the onsets of M2 related to M1, M3 related to M2, and M3 related to M1. **Table C**. ANOVA within each of the 19 elements. F and p values of ANOVA; post hoc analysis in case of significant values: Mean values, standard deviations (SD), Cohen’s *d*, and *p*-values for the temporal differences (T) of the onsets of M2 related to M1, M3 related to M2, and M3 related to M1. **Table D**. All counts and percentages of individual INOS patterns analyzed within the descriptive analysis.
(DOC)
