# Supplementary material for: The Punctum Fixum-Punctum Mobile Model: A Neuromuscular Principle for Efficient Movement Generation?
Source: PLoS One. 2015 Mar 30;10(3):e0120193. doi: 10.1371/journal.pone.0120193 (PMC4378895; doi:10.1371/journal.pone.0120193)
Supplement: S1 Dataset — All elements and trials per subject analyzed within the ANOVA. Table B. ANOVA within the six element groups. F and p values of ANOVA; post hoc analysis in case of significant values: Mean values, standard deviations (SD), Cohen’s d, and p-values for the temporal differences (T) of the onsets of M2 related to M1, M3 related to M2, and M3 related to M1. Table C. ANOVA within each of the 19 elements. F and p values of ANOVA; post hoc analysis in case of significant values: Mean values, standard deviations (SD), Cohen’s d, and p-values for the temporal differences (T) of the onsets of M2 related to M1, M3 related to M2, and M3 related to M1. Table D. All counts and percentages of individual INOS patterns analyzed within the descriptive analysis. (DOC) [file pone.0120193.s001.doc]

**S1_Dataset - Supporting Information**

**Table A in S1 file:** **Individual elements and trials.** All elements and trials per subject analyzed within the ANOVA.

| **Elements (No.)** | **Long swings (total n=118; counted INOS patterns* for the descriptive analysis: n=103)** | | | | | | | | | | | | | | | | |
| --- | --- | --- | --- | --- | --- | --- | --- | --- | --- | --- | --- | --- | --- | --- | --- | --- | --- |
| **Subjects** | S  1 | S  2 | S  3 | S  4 | S  5 | S  6 | S  7 | S  8 | S  9 | S  10 | S  11 | S  12 | S  13 | S  14 | S  15 | S  16 |  |
| **Rings** | **Elements on rings (in total: *n*=24; counted INOS patterns*: *n*=22)** | | | | | | | | | | | | | | | | **Sum** |
| Swings fwd (No. 1) |  | 4 |  |  |  |  |  |  |  |  |  |  | 4 |  |  |  | 8 |
| Felge (No.2) |  | 2 |  |  |  |  |  |  |  |  |  |  | 2 |  |  |  | 4 |
| Swings bwd (No. 3) |  | 4 |  |  |  |  |  |  |  |  |  |  | 4 |  |  |  | 8 |
| Uprise bwd (No. 4) |  | 2 |  |  |  |  |  |  |  |  |  |  | 2 |  |  |  | 4 |
|  |  |  |  |  |  |  |  |  |  |  |  |  |  |  |  |  | **24** |
| **High bar** | **Elements on high bar (in total: *n*=94)** | | | | | | | | | | | | | | | |  |
| GS bwd  (No. 5) |  |  |  |  |  |  |  |  |  |  |  |  |  | 10 | 10 | 10 | 30 |
| GS fwd  (No. 6) | 1 | 4 | 3 | 2 | 2 | 2 | 6 | 3 | 3 | 1 | 3 |  | 2 |  |  |  | 32 |
| Back-uprise (No. 7) | 5 |  |  |  |  |  |  |  |  |  |  |  | 6 |  |  |  | 11 |
| Vor/Mark (No. 8/9) |  |  |  | 1 |  |  |  |  | 2 |  | 1 |  |  |  |  |  | 4 |
| Dis.Streched (No. 10) | 3 |  |  |  |  | 3 | 2 |  |  |  |  |  |  |  |  |  | 8 |
| Dis. 2/1  (No. 11) |  |  | 2 | 2 |  | 1 |  | 2 |  |  |  |  |  |  |  |  | 7 |
| Dis. 2/2  (No. 12) |  |  |  |  |  |  |  |  | 2 |  |  |  |  |  |  |  | 2 |
|  |  |  |  |  |  |  |  |  |  |  |  |  |  |  |  |  | **94** |
| **High bar** | **Near axis swings (NAS: *n* in total=34; counted INOS patterns*: *n*=31)** | | | | | | | | | | | | | | | | |
| FreeHipCirc (No. 13) | 5 |  | 8 |  |  | 4 | 3 | 3 | 2 |  | 3 |  | 6 |  |  |  | **34** |
| **Parallel bars** | **Upper arm swings (UAS: *n* in total=15; counted INOS patterns*: *n*=15)** | | | | | | | | | | | | | | | | |
| Uprise bwd (No. 14) | 5 |  |  |  |  | 3 | 1 |  | 2 |  |  |  |  |  |  |  | 11 |
| Uprise fwd (No. 15) |  |  |  |  | 1 |  | 1 |  | 2 |  |  |  |  |  |  |  | 4 |
|  |  |  |  |  |  |  |  |  |  |  |  |  |  |  |  |  | **15** |
| **Parallel bars** | **Support swings (SupS: *n*=139; counted INOS patterns*: *n*=138)** | | | | | | | | | | | | | | | | |
| Sw. to Hdst (No. 16) | 10 |  |  |  | 15 | 24 | 30 | 10 | 15 | 7 |  |  |  |  |  |  | 111 |
| Salto bwd (No. 17) | 4 |  |  |  | 3 | 8 | 8 | 3 |  |  |  |  | 2 |  |  |  | 28 |
|  |  |  |  |  |  |  |  |  |  |  |  |  |  |  |  |  | **139** |
| **Trampoline** | **Handspring-Salto on Trampoline (HSS: total *n*=15; counted INOS patterns*: *n*=15)** | | | | | | | | | | | | | | | | |
| HSS  (No. 18) |  |  |  |  |  |  |  |  |  |  |  |  |  | 5 | 5 | 5 | **15** |
| **Floor** | **Pivot movements (Turns: total *n*=12; counted INOS patterns*: *n*=9)** | | | | | | | | | | | | | | | | |
| Turns fast (No. 19) | 1 |  | 1 | 2 | 1 | 1 | 1 | 2 | 1 | 1 |  | 1 |  |  |  |  | **12** |
| **Subjects** | S  1 | S  2 | S  3 | S  4 | S  5 | S  6 | S  7 | S  8 | S  9 | S  10 | S  11 | S  12 | S  13 | S  14 | S  15 | S  16 | Analyzed elements for ANOVA:  **=333**  Counted INOS patterns for descriptive analysis*:  **= 311** |

* The numbers described under “counted INOS patterns for the descriptive analysis” do not refer to the ANOVA, but to the descriptive analyses of individual INOS patterns (see methods). These numbers can be slightly lower, caused by trials that include “simultaneous” onsets (for further details, see methods).

**Table B in S1 file.** **ANOVA within the six element groups.** F and p values of ANOVA; post hoc analysis in case of significant values: Mean values, standard deviations (SD), Cohen’s *d*, and *p*-values for the temporal differences (T) of the onsets of M2 related to M1, M3 related to M2, and M3 related to M1.

| ANOVA | | | T: M1 (=0) to M2 | | | T: M2 to M3 | | | T: M1 (=0) to M3 | | |
| --- | --- | --- | --- | --- | --- | --- | --- | --- | --- | --- | --- |
| Element  groups | *F* | *p* | Mean±SD (s) | *d* | *p* | Mean±SD (s) | *d* | *p* | Mean±SD (s) | *d* | *p* |
| LS  *n=*118 | 92.989 | <0.001 | 0.048±  0.065 | 1.477 | <0.001 | 0.049±  0.066 | 0.742 | <0.001 | 0.097±  0.067 | 2.896 | <0.001 |
| NAS  *n*=34 | 33.875 | <0.001 | 0.008±  0.072 | 0.222 | 0.777 | 0.138±  0.098 | 1.408 | <0.001 | 0.147±  0.123 | 2.371 | <0.001 |
| UAS ^a^  *n*=15 | 3.397 | 0.043 |  |  |  | 0.035±  0.074 | 0.946 |  |  |  |  |
| SupS ^a^  *n*=139 | 234.963 | <0.001 |  |  |  | 0.078±  0.060 | 2.600 |  |  |  |  |
| HSS  *n*=15 | 27.670 | <0.001 | 0.079±  0.041 | 3.854 | <0.001 | 0.024±  0.048 | 0.500 | 0.378 | 0.103±  0.055 | 3.745 | <0.001 |
| Turns  *n*=12 | 7.240 | 0.002 | 0.017±  0.021 | 1.619 | 0.042 | 0.043±  0.044 | 0.977 | 0.113 | 0.060±  0.066 | 1.818 | 0.023 |
| All  *n=*333 | 261.088 | <0.001 | 0.022±  0.052 | 0.846 | <0.001 | 0.068±  0.064 | 1.063 | <0.001 | 0.090±  0.076 | 2.368 | <0.001 |

Abbreviations: LS: long swings; NAS: near axis swings; UAS: upper arm swings; SupS: support swings; HSS: take off during handspring fwd. – salto stretched fwd. on trampoline; Turns: fast pivot movements.

a: In upper arm swings and support swings, the shoulder axis is the rotational axis itself and the shoulder muscles have to stabilize this axis during these movements. Therefore, in these two movement groups the shoulder muscles were not included in the analysis.

**Table C in S1 file:** **ANOVA within each of the 19 elements**. F and p values of ANOVA; post hoc analysis in case of significant values: Mean values, standard deviations (SD), Cohen’s *d*, and *p*-values for the temporal differences (T) of the onsets of M2 related to M1, M3 related to M2, and M3 related to M1.

| ANOVA | | | T: M1 (=0) to M2 | | | T: M2 to M3 | | | T: M1 (=0) to M3 | | |
| --- | --- | --- | --- | --- | --- | --- | --- | --- | --- | --- | --- |
| Element numbers | *F* | *p* | Mean±SD (s) | *d* | *p* | Mean±SD (s) | *d* | *p* | Mean±SD (s) | *d* | *p* |
| 1/2  *n*=12 | 16.447 | <0.001 | 0.082±  0.059 | 2.780 | <0.001 | 0.019±  0.056 | 0.339 | 0.694 | 0.100±  0.053 | 3.774 | <0.001 |
| 3/4  *n*=12 | 4.326 | 0.021 | 0.054±  0.102 | 1.059 | 0.204 | 0.057±  0.113 | 0.504 | 0.450 | 0.111±  0.123 | 1.805 | 0.024 |
| 5  *n=*30 | 39.267 | <0.001 | 0.033±  0.035 | 1.886 | <0.001 | 0.041±  0.040 | 1.025 | 0.001 | 0.074±  0.044 | 3.364 | <0.001 |
| 6  *n*=32 | 26.364 | <0.001 | 0.047±  0.078 | 1.205 | 0.005 | 0.059±  0.072 | 0.819 | 0.005 | 0.106±  0.065 | 3.262 | <0.001 |
| 7  *n*=11 | 24.962 | <0.001 | 0.045±  0.053 | 1.698 | 0.047 | 0.065±  0.044 | 1.477 | 0.009 | 0.110±  0.035 | 6.286 | <0.001 |
| 8/9  *n*=4 | 45.409 | <0.001 | 0.045±  0.019 | 4.737 | 0.037 | 0.060±  0.019 | 3.158 | 0.010 | 0.105±  0.019 | 11.053 | 0.003 |
| 10/11/12  *n*=17 | 8.992 | 0.001 | 0.053±  0.065 | 1.631 | 0.019 | 0.045±  0.076 | 0.592 | 0.295 | 0.098±  0.087 | 2.253 | 0.004 |
| 13  *n*=34 | 33.875 | <0.001 | 0.008±  0.072 | 0.222 | 0.777 | 0.138±  0.098 | 1.408 | <0.001 | 0.147±  0.123 | 2.390 | <0.001 |
| 14 ^a^  *n*=11 | 0.073 | 0.930 |  |  |  | 0.004±  0.045 | 0.178 |  |  |  |  |
| 15 ^a^  *n*=4 | 11.488 | 0.003 |  |  |  | 0.123±  0.072 | 3.417 |  |  |  |  |
| 16 ^a^  *n*=111 | 158.081 | <0.001 |  |  |  | 0.074±  0.062 | 2.387 |  |  |  |  |
| 17 ^a^  *n*=28 | 100.834 | <0.001 |  |  |  | 0.096±  0.050 | 3.840 |  |  |  |  |
| 18  *n=*15 | 27.670 | <0.001 | 0.079±  0.041 | 3.854 | <0.001 | 0.024±  0.048 | 0.500 | 0.378 | 0.103±0.055 | 3.745 | <0.001 |
| 19  *n*=12 | 7.240 | 0.002 | 0.017±  0.021 | 1.619 | 0.042 | 0.043±  0.044 | 0.977 | 0.113 | 0.060±0.066 | 1.818 | 0.023 |

a: In upper arm swings and support swings, the shoulder axis is the rotational axis itself and the shoulder muscles have to stabilize this axis during these movements. Therefore, in these two movement groups the shoulder muscles were not included in the analysis.

**Table D in S1 file**: **Individual INOS patterns.** Individual counts and percentages per subject of INOS patterns for all singular and summarized elements of all the movement groups.* (Patterns without any counts are not listed).

| **Elements (No.)** | **Long swing elements (LS; INOS patterns analyzed: *n*=103)** | | | | | | | | | | | | | | | | |
| --- | --- | --- | --- | --- | --- | --- | --- | --- | --- | --- | --- | --- | --- | --- | --- | --- | --- |
| **Patterns** | **Counts of patterns: Long swing elements on rings (*n*=22)** | | | | | | | | | | | | | | | | |
| **Swings fwd** (No. 1) |  | | | | | | | | | | | | | | | |  |
|  | **Counts** | | | | | | | | | | | | | | | | **Sum** |
| **Subjects** | S  1 | S  2 | S  3 | S  4 | S  5 | S  6 | S  7 | S  8 | S  9 | S 10 | S  11 | S 12 | S  13 | S 14 | S 15 | S  16 |  |
| **1-2-3** |  | 4 |  |  |  |  |  |  |  |  |  |  | 1 |  |  |  | 5 |
| **2-1-3** |  |  |  |  |  |  |  |  |  |  |  |  | 1 |  |  |  | 1 |
| **1-3-2** |  |  |  |  |  |  |  |  |  |  |  |  | 2 |  |  |  | 2 |
|  |  |  |  |  |  |  |  |  |  |  |  |  |  |  |  |  | **8** |
|  | **Patterns in % of counts** | | | | | | | | | | | | | | | | **%** **in total** |
| **1-2-3** |  | 100 |  |  |  |  |  |  |  |  |  |  | 25 |  |  |  | 62.5 |
| **2-1-3** |  |  |  |  |  |  |  |  |  |  |  |  | 25 |  |  |  | 12.5 |
| **1-3-2** |  |  |  |  |  |  |  |  |  |  |  |  | 50 |  |  |  | 25 |
| **Felge**  (No. 2) |  | | | | | | | | | | | | | | | | |
|  | **Counts** | | | | | | | | | | | | | | | | |
| **1-2-3** |  | 2 |  |  |  |  |  |  |  |  |  |  |  |  |  |  | 2 |
| **1-3-2** |  |  |  |  |  |  |  |  |  |  |  |  | 2 |  |  |  | 2 |
|  |  |  |  |  |  |  |  |  |  |  |  |  |  |  |  |  | **4** |
|  | **Patterns in % of counts** | | | | | | | | | | | | | | | | **%** **in total** |
| **1-2-3** |  | 100 |  |  |  |  |  |  |  |  |  |  |  |  |  |  | 50 |
| **1-3-2** |  |  |  |  |  |  |  |  |  |  |  |  | 100 |  |  |  | 50 |
| **Swings bwd**  (No. 3) |  | | | | | | | | | | | | | | | | |
|  | **Counts** | | | | | | | | | | | | | | | | |
| **1-2-3** |  | 2 |  |  |  |  |  |  |  |  |  |  | 3 |  |  |  | 5 |
| **2-1-3** |  |  |  |  |  |  |  |  |  |  |  |  | 1 |  |  |  | 1 |
|  |  |  |  |  |  |  |  |  |  |  |  |  |  |  |  |  | **6** |
|  | **Patterns in % of counts** | | | | | | | | | | | | | | | | **%** **in total** |
| **1-2-3** |  | 100 |  |  |  |  |  |  |  |  |  |  | 75 |  |  |  | 87.5 |
| **2-1-3** |  |  |  |  |  |  |  |  |  |  |  |  | 25 |  |  |  | 12.5 |
| **Uprise bwd**  (No. 4) |  | | | | | | | | | | | | | | | | |
|  | **Counts** | | | | | | | | | | | | | | | | |
| **1-2-3** |  | 1 |  |  |  |  |  |  |  |  |  |  | 2 |  |  |  | 3 |
| **2-1-3** |  | 1 |  |  |  |  |  |  |  |  |  |  |  |  |  |  | 1 |
|  |  |  |  |  |  |  |  |  |  |  |  |  |  |  |  |  | **4** |
|  |  |  |  |  |  |  |  |  |  |  |  |  |  |  |  |  | **=22** |
|  | **Patterns in % of counts** | | | | | | | | | | | | | | | | **%** **in total** |
| **1-2-3** |  | 50 |  |  |  |  |  |  |  |  |  |  | 100 |  |  |  | 75 |
| **2-1-3** |  | 50 |  |  |  |  |  |  |  |  |  |  |  |  |  |  | 25 |
|  |  |  |  |  |  |  |  |  |  |  |  |  |  |  |  |  |  |
|  | **Long swings summarized on rings anterior muscle chain** | | | | | | | | | | | | | | | | |
| **Patterns** | **Counts** | | | | | | | | | | | | | | | | |
| **1-2-3** |  | 6 |  |  |  |  |  |  |  |  |  |  | 1 |  |  |  | 7 |
| **2-1-3** |  |  |  |  |  |  |  |  |  |  |  |  | 1 |  |  |  | 1 |
| **1-3-2** |  |  |  |  |  |  |  |  |  |  |  |  | 4 |  |  |  | 4 |
|  |  |  |  |  |  |  |  |  |  |  |  |  |  |  |  |  | **12** |
|  | **Patterns in % of counts** | | | | | | | | | | | | | | | | **%** **in total** |
| **1-2-3** |  | 100 |  |  |  |  |  |  |  |  |  |  | 16.66 |  |  |  | 58.33 |
| **2-1-3** |  |  |  |  |  |  |  |  |  |  |  |  | 16.66 |  |  |  | 8.33 |
| **1-3-2** |  |  |  |  |  |  |  |  |  |  |  |  | 66.66 |  |  |  | 33.33 |
|  | **Long swings summarized on rings posterior muscle chain** | | | | | | | | | | | | | | | | |
| **Patterns** | **Counts** | | | | | | | | | | | | | | | | |
| **1-2-3** |  | 3 |  |  |  |  |  |  |  |  |  |  | 5 |  |  |  | 8 |
| **2-1-3** |  | 1 |  |  |  |  |  |  |  |  |  |  | 1 |  |  |  | 2 |
|  |  |  |  |  |  |  |  |  |  |  |  |  |  |  |  |  | **10** |
|  | **Patterns in % of counts** | | | | | | | | | | | | | | | | **%** **in total** |
| **1-2-3** |  | 75 |  |  |  |  |  |  |  |  |  |  | 83.34 |  |  |  | 79.17 |
| **2-1-3** |  | 25 |  |  |  |  |  |  |  |  |  |  | 16.66 |  |  |  | 20.83 |
|  | **Long swings summarized on rings anterior and posterior muscle chain** | | | | | | | | | | | | | | | | |
| **Patterns** | **Counts** | | | | | | | | | | | | | | | | |
| **1-2-3** |  | 9 |  |  |  |  |  |  |  |  |  |  | 6 |  |  |  | 15 |
| **2-1-3** |  | 1 |  |  |  |  |  |  |  |  |  |  | 2 |  |  |  | 3 |
| **1-3-2** |  |  |  |  |  |  |  |  |  |  |  |  | 4 |  |  |  | 4 |
|  |  |  |  |  |  |  |  |  |  |  |  |  |  |  |  |  | **22** |
|  | **Patterns in % of counts** | | | | | | | | | | | | | | | | **%** **in total** |
| **1-2-3** |  | 90 |  |  |  |  |  |  |  |  |  |  | 49.98 |  |  |  | 69.99 |
| **2-1-3** |  | 10 |  |  |  |  |  |  |  |  |  |  | 16.66 |  |  |  | 13.34 |
| **1-3-2** |  |  |  |  |  |  |  |  |  |  |  |  | 33.32 |  |  |  | 16.67 |
|  | **Counts of patterns: Long swing elements on high bar (*n*=81)** | | | | | | | | | | | | | | | | |
| **Giant swings bwd**  (No. 5) |  | | | | | | | | | | | | | | | | **Sum** |
| **Patterns** | **Counts** | | | | | | | | | | | | | | | | |
| **1-2-3** |  |  |  |  |  |  |  |  |  |  |  |  |  | 8 | 9 | 10 | 27 |
| **1-3-2** |  |  |  |  |  |  |  |  |  |  |  |  |  |  | 1 |  | 1 |
| **2-3-1** |  |  |  |  |  |  |  |  |  |  |  |  |  | 2 |  |  | 2 |
|  |  |  |  |  |  |  |  |  |  |  |  |  |  |  |  |  | **30** |
|  | **Patterns in % of counts** | | | | | | | | | | | | | | | | **%** **in total** |
| **1-2-3** |  |  |  |  |  |  |  |  |  |  |  |  |  | 80 | 90 | 100 | 90 |
| **1-3-2** |  |  |  |  |  |  |  |  |  |  |  |  |  |  | 10 |  | 3.34 |
| **2-3-1** |  |  |  |  |  |  |  |  |  |  |  |  |  | 20 |  |  | 6.66 |
| **Giant swings fwd**  (No. 6) |  | | | | | | | | | | | | | | | |  |
| **Patterns** | **Counts** | | | | | | | | | | | | | | | | |
| **1-2-3** | 1 | 4 | 3 | 2 | 1 | 2 | 6 | 1 |  |  | 3 |  |  |  |  |  | 23 |
| **2-1-3** |  |  |  |  |  |  |  |  | 3 | 1 |  |  | 1 |  |  |  | 5 |
| **2-3-1** |  |  |  |  |  |  |  | 2 |  |  |  |  | 1 |  |  |  | 3 |
|  |  |  |  |  |  |  |  |  |  |  |  |  |  |  |  |  | **31** |
|  | **Patterns in % of counts** | | | | | | | | | | | | | | | | **%** **in total** |
| **1-2-3** | 100 | 100 | 100 | 100 | 100 | 100 | 100 | 33.33 |  |  | 100 |  |  |  |  |  | 69.44 |
| **2-1-3** |  |  |  |  |  |  |  |  | 100 | 100 |  |  | 50 |  |  |  | 20.83 |
| **2-3-1** |  |  |  |  |  |  |  | 66.67 |  |  |  |  | 50 |  |  |  | 9.73 |
| **Back-uprise**  (No. 7) |  | | | | | | | | | | | | | | | |  |
| **Patterns** | **Counts** | | | | | | | | | | | | | | | | |
| **1-2-3** | 2 |  |  |  |  |  |  |  |  |  |  |  | 1 |  |  |  | 3 |
| **2-1-3** |  |  |  |  |  |  |  |  |  |  |  |  | 1 |  |  |  | 1 |
| **1-3-2** | 1 |  |  |  |  |  |  |  |  |  |  |  |  |  |  |  | 1 |
|  |  |  |  |  |  |  |  |  |  |  |  |  |  |  |  |  | **5** |
|  | **Patterns in % of counts** | | | | | | | | | | | | | | | | **%** **in total** |
| **1-2-3** | 66.66 |  |  |  |  |  |  |  |  |  |  |  | 50 |  |  |  | 58.33 |
| **2-1-3** |  |  |  |  |  |  |  |  |  |  |  |  | 50 |  |  |  | 25 |
| **1-3-2** | 33.33 |  |  |  |  |  |  |  |  |  |  |  |  |  |  |  | 16.67 |
| **Voronin / Markelov**  (No. 8/9) |  | | | | | | | | | | | | | | | |  |
| **Patterns** | **Counts** | | | | | | | | | | | | | | | | |
| **1-2-3** |  |  |  | 1 |  |  |  |  | 1 |  | 1 |  |  |  |  |  | 3 |
| **2-3-1** |  |  |  |  |  |  |  |  | 1 |  |  |  |  |  |  |  | 1 |
|  |  |  |  |  |  |  |  |  |  |  |  |  |  |  |  |  | **4** |
|  | **Patterns in % of counts** | | | | | | | | | | | | | | | | **%** **in total** |
| **1-2-3** |  |  |  | 100 |  |  |  |  | 50 |  | 100 |  |  |  |  |  | 83.33 |
| **2-3-1** |  |  |  |  |  |  |  |  | 50 |  |  |  |  |  |  |  | 16.67 |
|  |  |  |  |  |  |  |  |  |  |  |  |  |  |  |  |  |  |
| **Dismount Streched**  (No. 10) |  | | | | | | | | | | | | | | | |  |
| **Patterns** | **Counts** | | | | | | | | | | | | | | | | |
| **1-2-3** | 3 |  |  |  |  | 3 | 1 |  |  |  |  |  |  |  |  |  | 7 |
| **2-1-3** |  |  |  |  |  | 1 |  |  |  |  |  |  |  |  |  |  | 1 |
|  |  |  |  |  |  |  |  |  |  |  |  |  |  |  |  |  | **8** |
|  | **Patterns in % of counts** | | | | | | | | | | | | | | | | **%** **in total** |
| **1-2-3** | 100 |  |  |  |  | 75 | 100 |  |  |  |  |  |  |  |  |  | 91.67 |
| **2-1-3** |  |  |  |  |  | 25 |  |  |  |  |  |  |  |  |  |  | 8.33 |
| **Dismount: Double soomersaults streched with 1 LAR****  (No. 11) |  | | | | | | | | | | | | | | | |  |
| **Patterns** | **Counts** | | | | | | | | | | | | | | | | |
| **1-2-3** |  |  | 2 |  |  |  |  |  |  |  |  |  |  |  |  |  | 2 |
|  |  |  |  |  |  |  |  |  |  |  |  |  |  |  |  |  | **2** |
|  | **Patterns in % of counts** | | | | | | | | | | | | | | | | **%** **in total** |
| **1-2-3** |  |  | 100 |  |  |  |  |  |  |  |  |  |  |  |  |  | 100 |
| **Dismount: Double soomersaults streched with 2 LAR****  (No. 12) |  | | | | | | | | | | | | | | | |  |
| **Patterns** | **Counts** | | | | | | | | | | | | | | | | |
| **1-2-3** |  |  |  |  |  |  |  |  | 1 |  |  |  |  |  |  |  | 1 |
|  |  |  |  |  |  |  |  |  |  |  |  |  |  |  |  |  | **1** |
|  |  |  |  |  |  |  |  |  |  |  |  |  |  |  |  |  | **= 81** |
|  | **Patterns in % of counts** | | | | | | | | | | | | | | | | **%** **in total** |
| **1-2-3** |  |  |  |  |  |  |  |  | 100 |  |  |  |  |  |  |  | 100 |
|  | **Long swings summarized on high bar anterior muscle chain** | | | | | | | | | | | | | | | | |
| **Patterns** | **Counts** | | | | | | | | | | | | | | | | |
| **1-2-3** | 4 | 4 | 5 | 2 | 1 | 5 | 7 | 1 | 1 |  | 3 |  |  | 8 | 9 | 10 | 60 |
| **2-1-3** |  |  |  |  |  | 1 |  |  | 3 | 1 |  |  | 1 |  |  |  | 6 |
| **1-3-2** |  |  |  |  |  |  |  |  |  |  |  |  |  |  | 1 |  | 1 |
| **2-3-1** |  |  |  |  |  |  |  | 2 |  |  |  |  | 1 | 2 |  |  | 5 |
|  |  |  |  |  |  |  |  |  |  |  |  |  |  |  |  |  | **72** |
|  | **Patterns in % of counts** | | | | | | | | | | | | | | | | **%** **in total** |
| **1-2-3** | 100 | 100 | 100 | 100 | 100 | 83.33 | 100 | 33.33 | 25 |  | 100 |  |  | 80 | 90 | 100 | 74.11 |
| **2-1-3** |  |  |  |  |  | 16.66 |  |  | 75 | 100 |  |  | 50 |  |  |  | 16.11 |
| **1-3-2** |  |  |  |  |  |  |  |  |  |  |  |  |  |  | 10 |  | 0.67 |
| **2-3-1** |  |  |  |  |  |  |  | 66.66 |  |  |  |  | 50 | 20 |  |  | 9.11 |
|  | **Long swings summarized on high bar posterior muscle chain** | | | | | | | | | | | | | | | | |
| **Patterns** | **Counts** | | | | | | | | | | | | | | | | |
| **1-2-3** | 2 |  |  | 1 |  |  |  |  | 1 |  | 1 |  | 1 |  |  |  | 6 |
| **2-1-3** |  |  |  |  |  |  |  |  |  |  |  |  | 1 |  |  |  | 1 |
| **1-3-2** | 1 |  |  |  |  |  |  |  |  |  |  |  |  |  |  |  | 1 |
| **2-3-1** |  |  |  |  |  |  |  |  | 1 |  |  |  |  |  |  |  | 1 |
|  |  |  |  |  |  |  |  |  |  |  |  |  |  |  |  |  | **9** |
|  | **Patterns in % of counts** | | | | | | | | | | | | | | | | **%** **in total** |
| **1-2-3** | 66.66 |  |  | 100 |  |  |  |  | 50 |  | 100 |  | 50 |  |  |  | 73.33 |
| **2-1-3** |  |  |  |  |  |  |  |  |  |  |  |  | 50 |  |  |  | 10 |
| **1-3-2** | 33.33 |  |  |  |  |  |  |  |  |  |  |  |  |  |  |  | 6.67 |
| **2-3-1** |  |  |  |  |  |  |  |  | 50 |  |  |  |  |  |  |  | 10 |
|  | **Long swings summarized on high bar anterior and posterior muscle chain** | | | | | | | | | | | | | | | | |
| **Patterns** | **Counts** | | | | | | | | | | | | | | | | |
| **1-2-3** | 6 | 4 | 5 | 3 | 1 | 5 | 7 | 1 | 2 |  | 4 |  | 1 | 8 | 9 | 10 | 66 |
| **2-1-3** |  |  |  |  |  | 1 |  |  | 3 | 1 |  |  | 2 |  |  |  | 7 |
| **1-3-2** | 1 |  |  |  |  |  |  |  |  |  |  |  |  |  | 1 |  | 2 |
| **2-3-1** |  |  |  |  |  |  |  | 2 | 1 |  |  |  | 1 | 2 |  |  | 6 |
|  |  |  |  |  |  |  |  |  |  |  |  |  |  |  |  |  | **81** |
|  | **Patterns in % of counts** | | | | | | | | | | | | | | | | **%** **in total** |
| **1-2-3** | 85.71 | 100 | 100 | 100 | 100 | 83.33 | 100 | 33.33 | 33.34 |  | 100 |  | 25 | 80 | 90 | 100 | 75.38 |
| **2-1-3** |  |  |  |  |  | 16.67 |  |  | 50 | 100 |  |  | 50 |  |  |  | 14.44 |
| **1-3-2** | 14.29 |  |  |  |  |  |  |  |  |  |  |  |  |  | 10 |  | 1.62 |
| **2-3-1** |  |  |  |  |  |  |  | 66.66 | 16.66 |  |  |  | 25 | 20 |  |  | 8.56 |
|  |  | | | | | | | | | | | | | | | | |
|  | **Long swings summarized all elements, anterior and posterior muscle chain (LS: *n*=103)** | | | | | | | | | | | | | | | | |
| **Patterns** | **Counts** | | | | | | | | | | | | | | | | |
| **1-2-3** | 6 | 13 | 5 | 3 | 1 | 5 | 7 | 1 | 2 |  | 4 |  | 7 | 8 | 9 | 10 | 81 |
| **2-1-3** |  | 1 |  |  |  | 1 |  |  | 3 | 1 |  |  | 4 |  |  |  | 10 |
| **1-3-2** | 1 |  |  |  |  |  |  |  |  |  |  |  | 4 |  | 1 |  | 6 |
| **2-3-1** |  |  |  |  |  |  |  | 2 | 1 |  |  |  | 1 | 2 |  |  | 6 |
|  |  |  |  |  |  |  |  |  |  |  |  |  |  |  |  |  | **103** |
|  |  | | | | | | | | | | | | | | | | |
|  | **Patterns in % of counts** | | | | | | | | | | | | | | | | **%** **in total** |
| **1-2-3** | 85.71 | 92.86 | 100 | 100 | 100 | 83.33 | 100 | 33.33 | 33.33 |  | 100 |  | 43.75 | 80 | 90 | 100 | 76.15 |
| **2-1-3** |  | 7.14 |  |  |  | 16.67 |  |  | 50 | 100 |  |  | 25 |  |  |  | 13.25 |
| **1-3-2** | 14.29 |  |  |  |  |  |  |  |  |  |  |  | 25 |  | 10 |  | 3.29 |
| **2-3-1** |  |  |  |  |  |  |  | 66.66 | 16.67 |  |  |  | 6.25 | 20 |  |  | 7.31 |
|  | **Near axis swings (NAS; INOS patterns analyzed: n=31)** | | | | | | | | | | | | | | | | |
|  | **Near axis swings summarized (NAS: *n*=31)** | | | | | | | | | | | | | | | | |
| **FreeHipCircle**  (No. 13) |  | | | | | | | | | | | | | | | | |
| **Patterns** | **Counts** | | | | | | | | | | | | | | | | |
| **1-2-3** | 4 |  | 2 |  |  |  |  |  | 2 |  |  |  | 2 |  |  |  | 10 |
| **2-1-3** | 1 |  | 4 |  |  | 2 | 3 | 2 |  |  | 2 |  | 4 |  |  |  | 18 |
| **1-3-2** |  |  |  |  |  | 1 |  |  |  |  | 1 |  |  |  |  |  | 2 |
| **2-3-1** |  |  |  |  |  | 1 |  |  |  |  |  |  |  |  |  |  | 1 |
|  |  |  |  |  |  |  |  |  |  |  |  |  |  |  |  |  | **31** |
|  | **Patterns in % of counts** | | | | | | | | | | | | | | | | **%** **in total** |
| **1-2-3** | 80 |  | 33.33 |  |  |  |  |  | 100 |  |  |  | 33.33 |  |  |  | 30.83 |
| **2-1-3** | 20 |  | 66.66 |  |  | 50 | 100 | 100 |  |  | 66.66 |  | 66.66 |  |  |  | 58.75 |
| **1-3-2** |  |  |  |  |  | 25 |  |  |  |  | 33.33 |  |  |  |  |  | 7.29 |
| **2-3-1** |  |  |  |  |  | 25 |  |  |  |  |  |  |  |  |  |  | 3.13 |
|  | **Upper arm swings (INOS patterns analyzed: *n*=15)** | | | | | | | | | | | | | | | | |
|  | **Upper arm swings posterior muscle chain** | | | | | | | | | | | | | | | | |
| **Uprise bwd**  (No. 14) |  | | | | | | | | | | | | | | | | |
| **Patterns** | **Counts** | | | | | | | | | | | | | | | | |
| **2-3** |  |  |  |  |  | 3 | 1 |  | 2 |  |  |  |  |  |  |  | 6 |
| **3-2** | 5 |  |  |  |  |  |  |  |  |  |  |  |  |  |  |  | 5 |
|  |  |  |  |  |  |  |  |  |  |  |  |  |  |  |  |  | **11** |
|  | **Patterns in % of counts** | | | | | | | | | | | | | | | | **%** **in total** |
| **2-3** |  |  |  |  |  | 100 | 100 |  | 100 |  |  |  |  |  |  |  | 75 |
| **3-2** | 100 |  |  |  |  |  |  |  |  |  |  |  |  |  |  |  | 25 |
|  | **Upper arm swings anterior muscle chain** | | | | | | | | | | | | | | | | |
| **Uprise fwd**  (No. 15) |  | | | | | | | | | | | | | | | | |
| **Patterns** | **Counts** | | | | | | | | | | | | | | | | |
| **2-3** |  |  |  |  | 1 |  | 1 |  | 2 |  |  |  |  |  |  |  | 4 |
|  |  |  |  |  |  |  |  |  |  |  |  |  |  |  |  |  | **4** |
|  | **Patterns in % of counts** | | | | | | | | | | | | | | | | **%** **in total** |
| **2-3** |  |  |  |  | 100 |  | 100 |  | 100 |  |  |  |  |  |  |  | 100 |
|  | **Upper arm swings summarized anterior and posterior muscle chain ( UAS: *n*=15)** | | | | | | | | | | | | | | | | |
| **Patterns** | **Counts** | | | | | | | | | | | | | | | | |
| **2-3** |  |  |  |  | 1 | 3 | 2 |  | 4 |  |  |  |  |  |  |  | 10 |
| **3-2** | 5 |  |  |  |  |  |  |  |  |  |  |  |  |  |  |  | 5 |
|  |  |  |  |  |  |  |  |  |  |  |  |  |  |  |  |  | **15** |
|  |  | | | | | | | | | | | | | | | | |
|  | **Patterns in % of counts** | | | | | | | | | | | | | | | | **%** **in total** |
| **2-3** |  |  |  |  | 100 | 100 | 100 |  | 100 |  |  |  |  |  |  |  | 80 |
| **3-2** | 100 |  |  |  |  |  |  |  |  |  |  |  |  |  |  |  | 20 |
|  | **Support swings posterior muscle chain** | | | | | | | | | | | | | | | | |
| **Swings to Hdst**  (No. 16) |  | | | | | | | | | | | | | | | | |
| **Patterns** | **Counts** | | | | | | | | | | | | | | | | |
| **2-3** | 10 |  |  |  | 15 | 21 | 30 | 10 | 15 | 6 |  |  |  |  |  |  | 107 |
| **3-2** |  |  |  |  |  | 3 |  |  |  |  |  |  |  |  |  |  | 3 |
|  |  |  |  |  |  |  |  |  |  |  |  |  |  |  |  |  | **110** |
|  | **Patterns in % of counts** | | | | | | | | | | | | | | | | **%** **in total** |
| **2-3** | 100 |  |  |  | 100 | 87.50 | 100 | 100 | 100 | 100 |  |  |  |  |  |  | 98.21 |
| **3-2** |  |  |  |  |  | 12.50 |  |  |  |  |  |  |  |  |  |  | 1.79 |
|  | **Support swings anterior muscle chain** | | | | | | | | | | | | | | | | |
| **Dismount: Salto streched bwd**  (No. 17) |  | | | | | | | | | | | | | | | | |
| **Patterns** | **Counts** | | | | | | | | | | | | | | | | |
| **2-3** | 4 |  |  |  | 3 | 8 | 8 | 3 |  |  |  |  | 2 |  |  |  | 28 |
|  |  |  |  |  |  |  |  |  |  |  |  |  |  |  |  |  | **28** |
|  | **Patterns in % of counts** | | | | | | | | | | | | | | | | **%** **in total** |
| **2-3** | 100 |  |  |  | 100 | 100 | 100 | 100 |  |  |  |  | 100 |  |  |  | 100 |
|  | **Support swings summarized anterior and posterior muscle chain (*n*=138)** | | | | | | | | | | | | | | | | |
| **Patterns** | **Counts** | | | | | | | | | | | | | | | | |
| **2-3** | 14 |  |  |  | 18 | 29 | 38 | 13 | 15 | 6 |  |  | 2 |  |  |  | 135 |
| **3-2** |  |  |  |  |  | 3 |  |  |  |  |  |  |  |  |  |  | 3 |
|  |  |  |  |  |  |  |  |  |  |  |  |  |  |  |  |  | **138** |
|  | **Patterns in % of counts** | | | | | | | | | | | | | | | | **%** **in total** |
| **2-3** | 100 |  |  |  | 100 | 90.63 | 100 | 100 | 100 | 100 |  |  | 100 |  |  |  | 98.83 |
| **3-2** |  |  |  |  |  | 9.38 |  |  |  |  |  |  |  |  |  |  | 1.17 |
|  | **Handspring-Salto on Trampoline (HSS; INOS patterns analyzed: *n*=15)** | | | | | | | | | | | | | | | | |
| **HSS**  (No. 18) | **Handspring-Salto on Trampoline (HSS: *n*=15)** | | | | | | | | | | | | | | | | |
| **Patterns** | **Counts** | | | | | | | | | | | | | | | | |
| **1-2-3** |  |  |  |  |  |  |  |  |  |  |  |  |  | 4 | 4 | 5 | 13 |
| **1-3-2** |  |  |  |  |  |  |  |  |  |  |  |  |  | 1 | 1 |  | 2 |
|  |  |  |  |  |  |  |  |  |  |  |  |  |  |  |  |  | **15** |
|  | **Patterns in % of counts** | | | | | | | | | | | | | | | | **%** **in total** |
| **1-2-3** |  |  |  |  |  |  |  |  |  |  |  |  |  | 80 | 80 | 100 | 86.67 |
| **1-3-2** |  |  |  |  |  |  |  |  |  |  |  |  |  | 20 | 20 |  | 13.33 |
|  |  | | | | | | | | | | | | | | | | |
|  | **Pivot movements (Turns: INOS patterns analyzed: *n=*9)** | | | | | | | | | | | | | | | | |
| **Turns**  (No. 19) | **Pivot movements (Turns: *n*=9)** | | | | | | | | | | | | | | | | |
| **Patterns** | **Counts** | | | | | | | | | | | | | | | | |
| **1-2-3** |  |  |  |  | 1 | 1 | 1 | 1 | 1 | 1 |  | 1 |  |  |  |  | 7 |
| **3-1-2** |  |  |  | 1 |  |  |  |  |  |  |  |  |  |  |  |  | 1 |
| **2-3-1** |  |  |  |  |  |  |  | 1 |  |  |  |  |  |  |  |  | 1 |
|  |  |  |  |  |  |  |  |  |  |  |  |  |  |  |  |  | **9** |
|  | **Patterns in % of counts** | | | | | | | | | | | | | | | | **%** **in total** |
| **1-2-3** |  |  |  |  | 100 | 100 | 100 | 50 | 100 | 100 |  | 100 |  |  |  |  | 81.25 |
| **3-1-2** |  |  |  | 100 |  |  |  |  |  |  |  |  |  |  |  |  | 12.5 |
| **2-3-1** |  |  |  |  |  |  |  | 50 |  |  |  |  |  |  |  |  | 6.25 |
| **Subjects** | S  1 | S  2 | S  3 | S  4 | S  5 | S  6 | S  7 | S  8 | S  9 | S 10 | S  11 | S 12 | S  13 | S 14 | S 15 | S  16 | Analyzed  in total  **=311 counts** |

* The numbers of trials within this analysis of individual INOS patterns is slightly lower than the numbers of trials the ANOVA (see Table A in S1 file). Some trials could not be surely matched to a specific pattern, because they include one or more “simultaneous” onsets. Onsets were defined as “simultaneous” when the time difference between onsets is less than 0.01 sec (see methods for further details).

** LAR: Longitudinal axis rotation
